# *ARID5B* regulates fatty acid metabolism and proliferation at the Pre-B cell stage during B cell development

**DOI:** 10.3389/fimmu.2023.1170475

**Published:** 2023-07-07

**Authors:** Jaya Prakash Chalise, Ali Ehsani, Mengistu Lemecha, Yu-Wen Hung, Guoxiang Zhang, Garrett P. Larson, Keiichi Itakura

**Affiliations:** ^1^ Center for RNA Biology and Therapeutics, Beckman Research Institute, City of Hope, Duarte, CA, United States; ^2^ Immunology and Theranostics, Beckman Research Institute, City of Hope, Duarte, CA, United States

**Keywords:** ARID5B, B cell development, pre-B cells, fatty acid metabolism, B-ALL

## Abstract

During B cell development in bone marrow, large precursor B cells (large Pre-B cells) proliferate rapidly, exit the cell cycle, and differentiate into non-proliferative (quiescent) small Pre-B cells. Dysregulation of this process may result in the failure to produce functional B cells and pose a risk of leukemic transformation. Here, we report that AT rich interacting domain 5B (*ARID5B*), a B cell acute lymphoblastic leukemia (B-ALL) risk gene, regulates B cell development at the Pre-B stage. In both mice and humans, we observed a significant upregulation of *ARID5B* expression that initiates at the Pre-B stage and is maintained throughout later stages of B cell development. In mice, deletion of *Arid5b in vivo* and *ex vivo* exhibited a significant reduction in the proportion of immature B cells but an increase in large and small Pre-B cells. *Arid5b* inhibition *ex vivo* also led to an increase in proliferation of both Pre-B cell populations. Metabolic studies in mouse and human bone marrow revealed that fatty acid uptake peaked in proliferative B cells then decreased during non-proliferative stages. We showed that *Arid5b* ablation enhanced fatty acid uptake and oxidation in Pre-B cells. Furthermore, decreased *ARID5B* expression was observed in tumor cells from B-ALL patients when compared to B cells from non-leukemic individuals. In B-ALL patients, *ARID5B* expression below the median was associated with decreased survival particularly in subtypes originating from Pre-B cells. Collectively, our data indicated that *Arid5b* regulates fatty acid metabolism and proliferation of Pre-B cells in mice, and reduced expression of *ARID5B* in humans is a risk factor for B cell leukemia.

## 1 Introduction

Everyday billions of B cells are produced in human bone marrow (BM) and play an integral role in immune functions such as antibody production, antigen presentation and cytokine production. Governed by transcription factors and cytokines, B cell development initiates from hematopoietic stem cells (HSC) and proceeds through a series of lineage-commitment stages that include multipotent progenitor’s cells (MPP), common lymphoid progenitor’s cells (CLP), progenitor B cells (Pro-B cells), precursor B cells (large and small Pre-B cells) and immature B cells (1). Immature B cells expressing B cell receptor (BCR) egress from the BM niche, and continue maturation in the spleen and lymph nodes generating B cell subsets of follicular B cells, marginal zone B cells and germinal center B cells (2).

B cell development involves dynamic shifts in proliferation patterns and metabolism at different stages (3–5). After successful V(D)J recombination for Ig-heavy chain, under the influence of IL-7 and pre-BCR signaling, large Pre-B cells undergo rapid proliferation from four to six cell divisions known as a proliferative burst (6–8). At this point, metabolism is very active as evidenced by increased glucose uptake, glycolysis and oxidative phosphorylation (OxPhos), generating energy and macromolecules necessary for cell replication (7, 9–11). After this burst, large Pre-B cells exit the cell cycle and differentiate to quiescent small Pre-B cells with dramatic reductions in glucose uptake, glycolysis and OxPhos (9–11). Several transcription factors such Ikzf1, Ikzf3, Foxo1 along with adaptor molecules such as Blnk that inhibit glucose metabolism are involved in this transition (12–19). Like other proliferating cells, Pre-B cells are also likely to utilize fatty acids and glutamine in addition to glucose metabolism; however, information about lipid metabolism during B lymphopoiesis is very limited.

Quiescence in the small Pre-B cell stage is a prerequisite for VJ recombination at the Igκ locus, producing a light chain that subsequently combines with the µ-H chain to form the BCR on immature B cells (8, 20, 21). Dysregulation in the Pre-B stage may have consequences not only in the production of functional B cells but also in the malignant transformation of Pre-B cells (22–26). Indeed, a large percentage of B-ALL subtypes are transformed at Pre-B stages, and the resultant leukemic cells mimic the proliferation signaling of the Pre-B cells (26, 27). Hence, it is important to decipher mechanistically how highly proliferative and metabolically active large Pre-B cells transit to quiescent small Pre-B cells and how this quiescence is maintained.

AT rich interacting domain (ARID) 5B is a transcription factor which was originally discovered in our laboratory by virtue of its interaction with the modulator region of the immediate early gene of the human cytomegalovirus (28–30). Subsequently, several Genome Wide Association Studies (GWAS) in ethnically diverse populations of B-ALL patients identified *ARID5B* as a risk gene (31). Reduced *ARID5B* expression in human BM is also associated with both disease incidence and relapse in B-ALL patients (32, 33). Studies in mice indicate that *Arid5b* influences growth, development and metabolism of several cell types including adipocytes, chondrocytes and muscle (34–37). In adipocytes, *Arid5b* inhibition decreases *Ppar-γ* expression and activates both lipolysis and triglyceride synthesis resulting in increased energy dissipation (38). Furthermore, in human natural killer cells and pre-adipocytes, ARID5B is reported to influence the glycolysis and mitochondrial OxPhos (39, 40). Similarly, we recently reported that *Arid5b*-deficient skeletal muscles exhibited increased glucose uptake and beta-oxidation of fatty acids (41).

Despite its association with B-ALL, the fundamental role(s) of ARID5B in B cell development and leukemic transformation remain vague. A limited study showed that 3-week-old *Arid5b* deficient mice exhibited a lower percentage of IgM^-^B220^+^ cells in BM suggesting a role of Arid5b in B cell development (42). This concept was corroborated in a very recent study performed in *Arid5b* overexpressing mice (43). Expanding on these findings, here we demonstrate that *Arid5b* expression is upregulated at the large Pre-B cell stage during B cell development and plays a role in limiting the proliferation of both large and small Pre-B cells facilitating their differentiation into immature B cells. In addition, we found that *Arid5b* inhibits fatty acid uptake and oxidation in Pre-B cells. Furthermore, we provide additional evidence that reduced *ARID5B* expression is directly associated with increased disease risk and mortality in pediatric B-ALL patients.

## 2 Materials and methods

### 2.1 Mice

*Arid5b ^fl/fl^* mice were generated as previously described (34). *Arid5b ^fl/fl^* mice were bred with wild type (Wt) C57B6/J mice to generate *Arid5b ^fl+/-^* mice which were further bred with Cre-deleter mice (*Hprt-Cre*) (44) to generate *Arid5b^+/-^* mice. These *Arid5b^+/-^* mice were bred to generate *Arid5b^-/-^* mice and their Wt littermates. We verified the lack of *Arid5b* expression in BM and spleen from *Arid5b^-/-^* mice by qRTPCR, (**Supplementary Figures 2A, B**). Mice were fed standard chow *ad libitum* and maintained under controlled 12 hour light– dark cycles. Ten to fifteen-week old mice were used for all experiments and these studies were approved by the City of Hope Institutional Animal Care and Use Committee.

### 2.2 Human bone marrow, apheresis blood samples and leukemic B cells samples

Human BM and apheresis specimens were collected from healthy donors registered at the City of Hope National Medical Center who had consented to an Institutional Review Board (IRB) approved protocol COH IRB#06229. Cells were kept in liquid nitrogen until used. Leukemic B cells Lax7, Lax2, Pdx2 were kindly provided by Dr. Markus Müschen, Yale University (27).

### 2.3 Mouse B cell isolation and culture

After euthanization, BM cells from tibia and femur were isolated as described by Amend et al, 2016 (45). B cells were separated using magnetic CD45R (B220^+^) microbeads (Miltenyi Biotech, Germany). B cells or whole BM cells were then cultured in alpha-MEM culture media without nucleosides (Thermofisher Scientific, Waltham, MA) containing 20% FBS, 100 IU/mL penicillin, 100 μg/mL streptomycin, 50μM β-mercaptoethanol in the presence of 10 ng/mL recombinant mouse IL-7 (Pepro Tech Inc., Cranbury, NJ, USA). For analysis of IgM^+^ B cells, IL-7 was depleted from the culture 48 hr prior to analyses. Cells were maintained at 37°C in a humidified incubator with 5% CO_2_.

### 2.4 Antibodies and flow cytometry

All fluorochrome conjugated antibodies used were obtained from Biolegend San Diego, CA, USA or Novus Biologicals, Centennial, CO, USA and are listed in **Supplementary Table 1**. BM cells or cultured B cells were washed with PBS and resuspended in FACS staining buffer (Biolegend). Cells were initially blocked with mouse or human CD16/32 for 5 mins and subsequently stained with antibodies according to the manufacturer’s instructions. After 1 hr or overnight incubation at 4° C, cells were washed with FACS buffer and analyzed with flow cytometer LSR Fortessa (BD Biosciences). For intracellular staining, after incubation with surface antibodies, the cells were fixed and permeabilized according to manufacturer’s directions (Biolegend). The cells were then stained with intracellular antibodies and incubated overnight at 4°C followed by washing and flow cytometry. Data were analyzed with FCS Express 7 software (*De Novo* Software, Glendale, CA). The gating strategies are presented in **Supplementary Figures 5**-**8**.

### 2.5 Mouse and human B cell sorting

For mouse, B cells (B220^+^) were isolated from BM as described above. Cells were blocked with CD16/32 for 5 min in 4°C and stained with Pro- and Pre-B markers for 30 min followed by washing with FACS buffer and were sorted by BD FACS Aria III. Human BM samples were similarly blocked, stained with markers for Pro-B cells and total Pre-B cells, and sorted by BD FACS Aria III. The gating strategies are presented in **Supplementary Figures 5** and **9**.

### 2.6 *In vitro* B cell differentiation from embryonic stem cells

CD19^+^ B cells were differentiated from mouse embryonic stem cells (ESC) *in vitro* as described (46). Briefly, at day 1, OP9 feeder cells (ATCC, Manassas, VA, USA) were cultured for 2-3 days until 80% confluent and ESC(R1) cells (ATCC) were added to this feeder layer in presence of 5 ng/mL recombinant human Flt3-L (R&D systems, Minneapolis, MN, USA). At day 5, the semi-adherent cells were transferred to a new plate with fresh OP9 cells and further cultured in the presence of 5 ng/mL FLT3-L and 5 ng/mL IL-7 (Peprotech). Starting on day 12, the non-adherent cells were transferred to fresh feeder cells with media and cytokines at a regular 3-4 day intervals. CD19^+^CD45^+^ B cell populations were observed from day 12 onward and reached up to 65% by day 24 (**Supplementary Figure 1C**).

### 2.7 Retrovirus production

Retroviral constructs MSCV ERT2 GFP-Puro, MSCV Cre-ERT2 GFP-Puro, pHIT160 (gagpol) and pHIT123 (ecotropic env) were kindly provided by Dr. Markus Müschen (27). Retroviral supernatants were produced by co-transfecting HEK 293FT cells with plasmids pHIT60 and pHIT123 using Lipofectamine 3000 kit (Thermofisher). Cells were cultured in high-glucose Dulbecco’s modified Eagle’s medium (DMEM, Invitrogen) containing 10% fetal bovine serum, 100 IU/ml penicillin, 100 μg/ml streptomycin, 25 mmol/l HEPES. After 24 hr, media was replaced with collection media containing 10% fetal bovine serum, 100 IU/ml penicillin, 100 μg/ml streptomycin, and 1% ViralBoost Reagent (Alstem, USA). Retroviral supernatants were collected 24, 48 and 72 hr after the addition of collection media and filtered through a 0.45-μm filter.

### 2.8 *Ex vivo Arid5b* deletion

*Arid5b* was deleted *ex vivo* in BM B cells by utilizing *Arid5b ^fl/fl^* mice and Cre mediated retroviral transduction (**Supplementary Figure 2E**). Briefly, viral supernatants containing ERT2 GFP-Puro or Cre-ERT2 GFP-Puro were loaded onto non-treated six-well culture plates coated with 50 μg ml^−1^ RetroNectin (Takara Bio, San Jose, CA) and centrifuged at 2,000xG for 90 min). B220^+^ B cells isolated from the BM were plated on the virus coated plates and centrifuged for 30 minutes at 600xG. Plates containing B cells were further incubated for 24-48 hr in the presence of 10 μg/ml IL-7 (PeproTech). Cre was activated on transduced cells by the addition of 1 μM 4-hydroxytamoxifen (4-OHT) (PeproTech). The transduction efficiency was quantified by flow cytometric analysis of GFP^+^ cells and *Arid5b* deletion was verified by qRTPCR analysis (**Supplementary Figures 2F, G**).

### 2.9 qRTPCR

RNA was isolated using the RNeasy Mini kit (Qiagen, Hilden, Germany) and cDNA was prepared using iScript™ Reverse Transcription Supermix, (BioRad, Hercules, CA, USA) according to the manufacturer’s instructions. qRTPCR was carried out using TaqMan™ Fast Advanced Master Mix (Thermofisher Scientific, Waltham, MA, USA). The PCR probes used are listed in **Supplementary Table 2**. The comparative delta (ΔΔ) Ct method normalized to β*-Actin* was used to determine the relative expression value.

### 2.10 Western blot

Cellular proteins were extracted using RIPA lysis buffer (Santa Cruz Biotechnologies, Dallas, USA) with the addition of PMSF, Sodium orthovanadate, and protease inhibitor (Santa Cruz Biotechnologies). Cell lysates were prepared in 1X Laemmli buffer and run on a 5-20% SDS PAGE gels (Nacalai Tesque, Kyoto, Japan). Proteins were transferred to PVD nitro-cellulose membranes using Trans-Blot^®^ Turbo™ Transfer System (Bio-Rad). Membranes were blocked with 5% milk for 1-2 hr and incubated with primary antibodies which were anti-G6pd (ab87230, Abcam, Cambridge, United Kingdom), anti-Mpc1 (14462, Cell Signaling Technology, Danvers, MA), anti-Mpc2 (46141 Cell Signaling Technology) and anti-β-Actin (5125, Cell Signaling Technology) overnight at 4^˚^C. After incubation with secondary antibody, the membrane was developed using chemo-luminance reagents Piece ECL Pierce™ ECL Western Blotting Substrate (Thermofisher). The luminescence was read using a luminescent image analyzer and densitometry analysis was performed with Bio-Rad Image Lab Software.

### 2.11 Seahorse metabolic assays

B cells were plated on XF96-well plates (Agilient Technologies, Santa Clara, CA, USA) at 3x10^5^ cells/well. The plate was centrifuged at 300xG for 3 min and both extra cellular acidification rate (ECAR) and oxygen consumption rate (OCR) were analyzed using a Seahorse XFe96 Flux Analyzer according the manufacturer’s instructions (Agilent Technologies). For oxidative pathway inhibition, the cells were treated with 10 µM etomoxir (Med Chem Express, NJ USA), 10 µM UK5099 (Tocris Bioscience, Bristol, UK) and 5 µM BPTES (Cayman Chemical, Ann Harbour, MI, USA) 15 minutes prior to the Seahorse assay. Parameters of glycolysis, glycolytic capacity, glycolytic reserve, basal respiration, ATP linked respiration and maximal respiration were calculated according to the manufacturer’s manual. Palmitate oxidation assays were performed with the Seahorse XF Palmitate-BSA FAO Substrate kit (Agilient Technologies) according to the manufacturer’s instructions. Briefly, cells were cultured for 6 hrs in substrate limited media, Palmitate or BSA was added 15 min prior to OCR analysis. Data were normalized to cell counts.

### 2.12 Glucose and palmitate uptake assays

Glucose uptake was measured *ex-vivo* in cultured B cells in the presence of 10 µg/ml IL-7 by using Glucose Uptake-Glo™ Assay kit (Promega, Madison, WI, USA) according to manufacturer’s instructions. Palmitate uptake was measured *ex vivo* in cultured B cells from mice or humans in the presence of 10 µg/ml IL-7. Palmitate-BODIPY (1µM, Thermofisher Scientific) was added to the culture and incubated for 3 hr in a cell culture incubator. The cells were subsequently washed three times with ice-cold PBS containing 2% FBS prior to flow cytometry as previously described and mean florescence intensity (MFI) was quantified.

### 2.13 Public database analysis

For analyzing *ARID5B* expression at various stages during B cell development, we utilized data from Gene Expression Omnibus (GEO) GSE77098, GSE100738 (47, 48) and from *Tabula Muris* database (https://tabula-muris.ds.czbiohub.org/) (49) for mouse and GSE24759 (50) for human. We extracted *ARID5B* expression data from B-ALL patients and CD19^+^B cells from healthy donors from the St. Jude PeCan Portal (https://proteinpaint.stjude.org/panall.html). We analyzed Kaplan Meyer survival curves from PeCan portal where B-ALL patients were partitioned into two groups based on *ARID5B* expression values above or below the median of the group. For comparison of *ARID5B* expression in low-risk vs high risk pediatric B-ALL patients, we analyzed RNA expression values as described (51). The RNA-Seq data are available in the European Genome-Phenome Archive (EGA) under accession number EGAS00001003266.

### 2.14 Statistical analysis

All graphs and statistical analyses were performed by Graph Pad Prism 7.0 software (Graph Pad Prism Software Inc., San Diego, CA). The comparison between two means was analyzed by a two-tailed unpaired Student’s *t*-test. P values less than 0.05 were considered as statistically significant. Data unless mentioned otherwise are represented as mean and standard error of mean (Mean ± SEM).

## 3 Results

### 3.1 *ARID5B* is predominantly upregulated at Pre-B cell stages and onwards during B cell development

**Figure 1:**
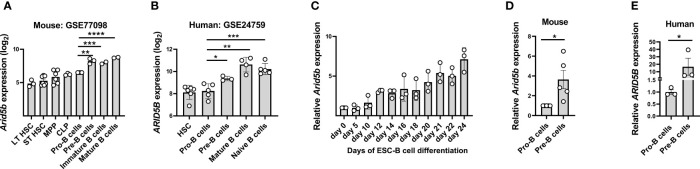
*ARID5B* expression is upregulated at the Pre-B cell stage during B cell development in mice and humans. **(A)** *Arid5b* expression in different stages of B cell development in mice, (GEO Accession no: GSE77098). n=3-6. **(B)** *ARID5B* expression in different stages of B cell development in humans, (GEO Accession no: GSE24759). n=3-6. **(C)** Relative *Arid5b* expression in cells collected at multiple time points during the differentiation of embryonic stem cells (ESC) to CD19^+^B cells *in vitro*. n=3. **(D)** Relative *Arid5b* expression in sorted Pro-B cells (CD19^+^cKIT^+^BP1^-^IgM^-^) and Pre-B cells (CD19^+^BP1^+^cKIT^-^IgM^-^) from mouse BM samples. n=5. **(E)** Relative *ARID5B* expression in sorted human Pro-B cells (CD10^+^CD19^-^CD34^+^) and Pre-B cells (CD10^+^CD19^+^IgM^-^). n=3. *β-Actin* was used as a normalizer for Figure C, D and **(E)** Data are shown with SEM and were analyzed by Student’s t test *p < 0.05, **p < 0.01, ***p < 0.001, ****p < 0.0001.

*ARID5B* is associated with B-ALL, a disease driven by defects in B cell development which is a multi-stage process tightly regulated at the transcriptional level; however, *ARID5B* expression during different stages of B cell development is unknown (31). Therefore, we first analyzed the expression of *Arid5b*/*ARID5B* in various cell stages of B cell development utilizing public databases of RNA expression both in mice and humans. In mouse BM, *Arid5b* expression increased gradually from hematopoietic stem cells (HSC) to Pro-B cells, sharply upregulated at the Pre-B cell stage and then remained stable at later stages of B cell development (**Figure 1A** and **Supplementary Figures 1A, B**). In humans, we observed similar B cell kinetics, as *ARID5B* expression was predominantly upregulated from Pre-B cells onwards (**Figure 1B**).

To confirm the upregulation of *Arid5b* during B cell development *in vitro*, mouse embryonic stem cells (ESC) were differentiated into B cells (CD45^+^CD19^+^) by co-culturing them on OP9 feeder cells in presence of FLT3 and IL-7 for 24 days (See methods and **Supplementary Figure 1C**). During this differentiation from ESC to B cells, we observed a gradual increase of *Arid5b* expression from day 0 to day 24 (**Figure 1C**). Next, we compared the *Arid5b* expression in sorted Pro-B cells (CD19^+^cKIT^+^BP1^-^IgM^-^) and total Pre-B cells (CD19^+^BP1^+^cKIT^-^IgM^-^) from BM of wild type (Wt) mice and found significantly higher *Arid5b* expression in total Pre-B cells compared to Pro-B cells (**Figure 1D**). To confirm if this trend is similar in humans, we measured the *ARID5B* expression in Pro-B cells (CD10^+^CD19^-^CD34^+^) and Pre-B cells (CD10^+^CD19^+^IgM^-^) from the BM of healthy donors. In agreement with the mouse data, human *ARID5B* expression was higher in the Pre-B cells compared to Pro-B cells (**Figure 1E**).

Overall, these findings show that the expression of *Arid5b*/*ARID5B* in both mouse and human was significantly upregulated during B cell development, particularly from Pro- to Pre-B cell stage suggesting a role at later stages of B cell lymphopoiesis.

### 3.2 *Arid5b* deficiency impairs B cell development at Pre-B cell stage

**Figure 2:**
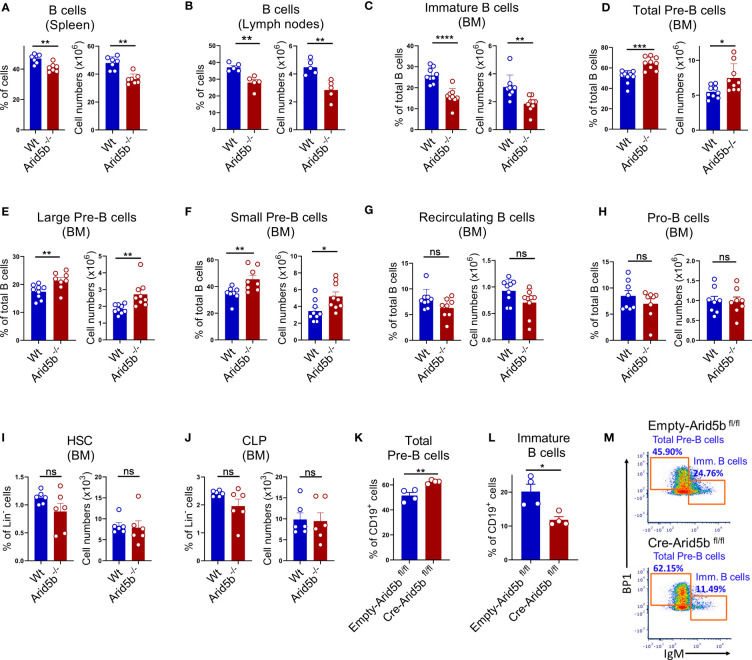
*Arid5b* deficiency impairs B cell development at Pre-B cell stage. **(A, B)** B cell populations of (B220^+^CD19^+^) in spleen and lymph nodes of Wt and *Arid5b^-/-^* mice. Data are expressed as percentage of B220^+^CD19^+^ cells out of total live cells (left bar graph) and absolute numbers per mice (right bar graph). n=5-7. **(C-H)** Populations of B lineage cells in BM of Wt and *Arid5b* ^-/-^ mice. Data are expressed as percentage of specific lineage cells out of total B cells (B220^+^CD19^+^ cells) (left bar graph) and absolute numbers per mice (right bar graph). n=8-9. **(I-J)** Population of HSC and CLP in BM of Wt and *Arid5b* ^-/-^ mice. Data are expressed as percentage of specific lineage cells out Lin^-^ cells (left bar graph) and absolute numbers per mice (right bar graph). n=6. **(K-M)** Sorted B cells from BM of *Arid5b ^fl/fl^* mice were retrovirally transduced with inducible Cre recombinase *ex vivo.* The figure represents the bar diagram **(K, L)** and a representative flow cytometry plot **(M)** showing the populations of total Pre-B and immature B cells 48 hrs after Cre-activation. n=4. Statistical data are shown with SEM error bars and were analyzed by Student’s t test. *p < 0.05, **p < 0.01, ***p < 0.001, ****p < 0.0001. ns, not significant.

To reveal the influence of *Arid5b* expression on B cell development, we compared *Arid5b* null (*Arid5b^-/-^*) mice with Wt littermate controls. Using flow cytometry to examine B cell populations in the spleen and lymph nodes, we found that the population of total B cells (B220^+^CD19^+^) was significantly lower in *Arid5b^-/-^* mice compared to the control mice indicating a defect in B cell development (**Figures 2A, B**). To determine which stage of B cell development is impaired in these mice, we investigated the populations of distinct B cell stages in BM. A significant reduction in the proportion of immature B cells (B220^+^CD19^+^IgM^+^IgD^-^) was observed in *Arid5b^-/-^* mice compared to Wt controls (**Figure 2C**). Interestingly, the proportions of total Pre-B cells (B220^+^CD19^+^BP1^+^cKIT^-^IgM^-^) consisting of large (B220^+^CD19^+^BP1^+^cKIT^-^IgM^-^FSC^high^) and small (B220^+^CD19^+^BP1^+^cKIT^-^IgM^-^FSC^low^) Pre-B cells were higher in *Arid5b^-/-^* mice compared to the controls (**Figures 2D-F**). However, we did not observe a significant difference in the populations of recirculating B cells (B220^+^CD19^+^IgM^+^IgD^+^), Pro-B cells (B220^+^CD19^+^cKIT^+^BP1^-^IgM^-^), HSC (Lin^-^Sca^+^cKIT^+^CD34^+^CD150^+^), and CLP (Lin^-^Sca^+^cKIT^+^CD34^+^IL-7R^+^) in the BM of *Arid5b^-/-^* and control mice (**Figures 2G-J**). These data indicate that B cell development is impaired in *Arid5b^-/-^* mice at the Pre-B stage. In these mice, the populations of T cells (CD45^+^CD3^+^) and the myeloid cells (CD45^+^CD11b^+^) were not altered in spleen and BM compared to Wt controls (**Supplementary Figures 2C, D**).

To determine if the effect is specific to B cell lineage but not of embryonic origin, we employed an *ex-vivo* model, whereby we isolated B cells (B220^+^cells) from the BM of *Arid5b ^flox/flox^* mice and retrovirally transduced these cells with an inducible *Cre* gene resulting in the deletion of *Arid5b* (**Supplementary Figures 2E-G**). Forty-eight hours after Cre activation, we analyzed total Pre-B cells (CD19^+^BP1^+^cKIT^-^IgM^-^) and immature B cells (CD19^+^IgM^+^cKIT^-^BP1^-^). Similar to our *in vivo* observations in *Arid5b^-/-^* mice, deletion of *Arid5b ex-vivo* increased the population of total Pre-B cells while preventing Pre-B cells from differentiating to immature cells (**Figures 2K-M**). Overall, these data indicated that *Arid5b* deficiency partially halted B cell development at the Pre-B cell stage impairing the differentiation to immature B cells.

### 3.3 *Arid5b* deficiency increases the proliferation of Pre-B cells perturbing the quiescence of small Pre-B cells

**Figure 3:**
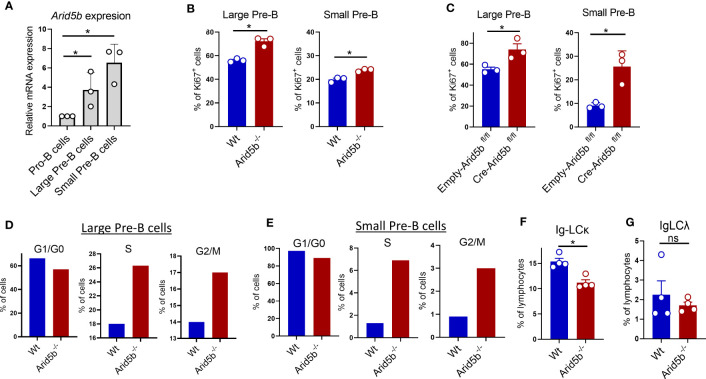
*Arid5b* deficiency enhances the proliferation of Pre-B cells. **(A)** *Arid5b* expression in sorted Pro-B cells (CD19^+^cKIT^+^BP1^-^IgM^-^), large Pre-B cells (CD19^+^BP1^+^cKIT^-^IgM^-^FSC^high^) and small Pre-B cells (CD19^+^BP1^+^cKIT^-^IgM^-^FSC^low^) from BM of Wt mice. n=3. **(B)** B220**^+^** B cells from BM were isolated from Wt and *Arid5b^-/-^* mice and cultured in the presence of IL-7. On day 3, cells were harvested, and stained with Ki67 and Pre-B cells markers followed by flow cytometry. The data represent the percentage of Ki67 positive large (CD19^+^BP1^+^cKIT^-^IgM^-^FSC^high^) and small (CD19^+^BP1^+^cKIT^-^IgM^-^FCS^low^) Pre-B cells. n=3. **(C)** B cells from BM were isolated from *Arid5b ^fl/fl^* mice, transduced with *Cre*-retrovirus (Cre-ERT2) or Control (Empty-ERT2). After 24 hrs in cell culture in the presence of IL-7, Cre was activated by 4-OHT and after an additional 48 hrs of culture, the cells were analyzed for Ki67^+^ as in **(B)**. n=3. **(D, E)** Cell cycle analyses by flow cytometry calculated based on DAPI staining on Wt and *Arid5b^-/-^* cells collected at day 3 as in **(B)**. The experiment was repeated three times with similar results. **(F, G)** Ig light chain kappa expression (Ig-LCκ^+^CD19^+^) and Ig light chain lamda expression (Ig-LCΛ^+^CD19^+^) analyzed by flow cytometry in Wt and *Arid5b^-/-^* BM cells. n=4. Data are shown with SEM and were analyzed by Student’s t test *p < 0.05. ns, not significant.

Pre-B cell stage begins with a proliferating phase (large Pre-B), followed by cell cycle exit and differentiation to a non-proliferating quiescent phase (small Pre-B) (8, 20). Upregulation of *Arid5b* initiated from the large Pre-B cells and continued to small Pre-B cells in mice (**Figure 3A**). We hypothesized that higher expression of *Arid5b* is required for cell cycle exit of proliferating large Pre-B cells and the maintenance of quiescence in small Pre-B cells. Therefore, deletion of *Arid5b* is expected to enhance the proliferation of Pre-B cells and perturb the quiescence of small Pre-B cells. To test this, we isolated B cells from the BM of Wt and *Arid5b^-/-^* mice, cultured them in the presence of IL-7 for 72 hrs, and stained with Ki67, an intracellular protein marker used to estimate proliferation (52). In comparison to Wt cells, both *Arid5b^-/-^* large and small Pre-B cells demonstrated a higher percentage of Ki67 positive cells (**Figure 3B** and **Supplementary Figures 3A, B**). As expected, a higher percentage of large Pre-B cells were Ki67 positive than small Pre-B cells (**Figure 3B** and **Supplementary Figures 3A, B**). A similar result of *Arid5b* deficient large and small Pre-B cells was observed in a *Cre*-inducible *Arid5b* knock out system *ex-vivo* (**Figure 3C**). These data indicate that *Arid5b* deletion enhances the proliferation of both large and small Pre-B cells and consequently disturbs the quiescence of small Pre-B cells. To further confirm the effect of *Arid5b* deletion in proliferation and quiescence, we performed cell cycle analyses. Compared to Wt, a higher percentage of *Arid5b* ^-/-^ large and small Pre-B cells were in S and G2/M phases indicating increased proliferation (**Figures 3D, E**). Consistently, the percentage of cells in G1/G0 phase in small Pre-B cells was reduced from 98% of cell population in WT to 88% *via Arid5b* deletion indicating the perturbation of quiescence (**Figure 3E**). A similar trend of cell cycle alterations was observed in an *Arid5b ex vivo* inducible knock out system (**Supplementary Figures 3C, D**). Maintenance of quiescence in small Pre-B cells is required for successful completion of VJ recombination at the *Igκ* and ***Igλ L*** locus to produce a light chain of the BCR (8, 20, 21). We observed a decreased population of B cells expressing Ig-LCκ but not Ig-LC*λ* in *Arid5b* deficient mice compared to Wt mice indicating an impairment in light chain recombination at the *Igκ L locus* (**Figures 3F, G**).

Along with the increased proliferation in *Arid5b^-/-^* large and small Pre-B cells, we observed an increase in *Myc* and *Myb* expression, encoding two transcription factors previously shown to facilitate proliferation of Pre-B cells in BM (**Supplementary Figures 3E, F**) (12, 53). However, no differences were observed in *Arid5b^-/-^* Pre-B cells for the expression of *Foxo1, Blnk, Ikzf1, Foxo3 and Fnip1*, factors known to regulate proliferation during B cell development (**Supplementary Figures 3G-K**).

These data show that *Arid5b* deficiency increases the proliferation of Pre-B cells and perturbs quiescence in small Pre-B cells which may explain impaired light chain recombination and the decreased proportion of immature B cells. These findings support our hypothesis: higher expression of *Arid5b* is required for cell cycle exit of proliferating large Pre-B cells and for the maintenance of quiescence in small Pre-B cells during B cell development.

### 3.4 *Arid5b* regulates fatty acid oxidation in Pre-B cells

**Figure 4:**
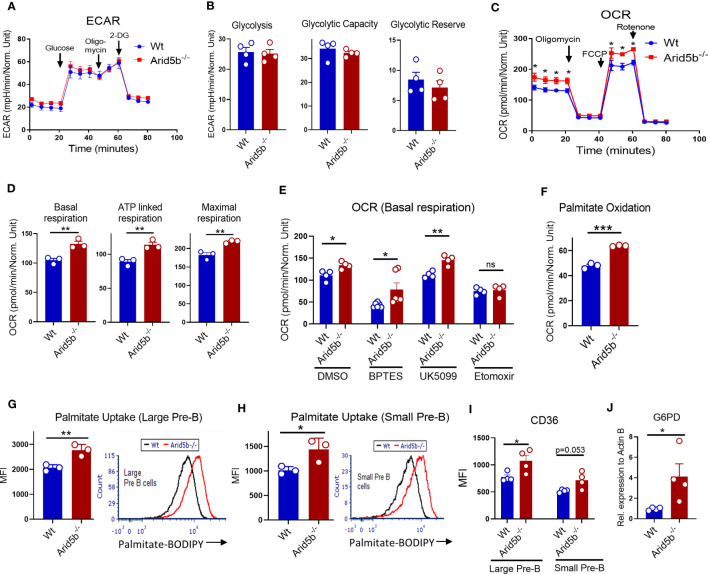
*Arid5b* downregulates fatty acid metabolism in Pre-B cells. **(A)** Seahorse ECAR analysis in BM B cells cultured in presence of IL-7 for 72 hr. Representative Seahorse trace of ECAR analysis. n=4 in each group. **(B)** Parameters of glycolysis, glycolytic capacity and glycolytic reserve were calculated from ECAR values after treatment with glucose, oligomycin and 2-DG. **(C)** OCR analysis of BM B cells cultured in presence of IL-7 for 72 hr. Representative Seahorse trace of OCR analysis. n=4 in each group. **(D)** Basal respiration, ATP linked respiration and maximal respiration were calculated by OCR values after treatment with oligomycin, FCCP and rotenone. **(E)** Basal respiration (OCR) values of Wt and *Arid5b^-/-^* Pre-B cells in the presence of glutaminase inhibitor (BPTES), Mpc1 inhibitor (UK5099), and CPT1 inhibitor (etomoxir). n=4. **(F)** Palmitate oxidation in Wt and *Arid5b^-/-^* cells. BM B cells were isolated and cultured in the presence of IL-7 and after three days the cells were cultured for 6 hours in substrate limited media. After the addition of palmitate, OCR was measured by a Seahorse analyzer. n=3. **(G, H)** Bar graph (left) quantifies the average mean fluorescence intensity (MFI) of palmitate-BODIPY FL C16 uptake in large Pre-B cells **(G)** and small Pre-B cells **(H)** from Wt and *Arid5b^-/^* ^-^ cells. n=3. Histograms (right) are representative flow cytometry MFI data for Wt (black line) and *Arid5b^-/^* ^-^ (red line) cells. **(I)** Flow cytometry analysis of surface expression of CD36 in large and small Pre-B cells. n=4. **(J)** Relative G6pd protein expression normalized to β-Actin expression by western blot in *ex vivo Arid5b* deleted Pre-B cells. n=4. All Seahorse experiments were repeated three times with similar results. Statistical data are shown with SEM and were analyzed by Student’s t test. *p < 0.05, **p < 0.01, ***p < 0.001.

*Arid5b* is known as a regulator of metabolism in pre-adipocytes, adipocytes, muscles and NK cells affecting glycolysis and oxidative phosphorylation (35, 38–40, 54). To investigate the effect of *Arid5b* ablation on metabolism of Pre-B cells, we isolated B220^+^B cells from BM, cultured in the presence of IL-7 for 72 hours, and measured extracellular acidification rate (ECAR) and oxidation consumption ratio (OCR) using a Seahorse metabolic flux analyzer. These cells consist of more than 75% CD19^+^BP1^+^ population, hence we considered these cells as Pre-B cells (**Supplementary Figure 4A**). As shown in **Figures 4A, B**, there were no significant differences in ECAR and glycolytic parameters (glycolysis, glycolytic-capacity and glycolytic reserve) between Wt and *Arid5b^-/-^* Pre-B cells. On the other hand, the OCR including basal, ATP-linked and maximal respiration were significantly increased in *Arid5b* deficient Pre-B cells (**Figures 4C, D**). These data indicated that glycolysis did not change but OxPhos was increased in *Arid5b^-/-^* Pre-B cells relative to Wt. Similar results were observed when *Arid5b* was ablated in Pre-B cells *ex-vivo* by retroviral transduction with Cre (**Supplementary Figure 4B**).

To identify which substrate(s) increased OxPhos in *Arid5b^-/-^* Pre-B cells, we measured OCR of Pre-B cells in the presence of specific chemical inhibitors for oxidation of glutamine, glucose and lipid. Upon treatment with the glutaminase inhibitor BPTES, the OCR in both Wt and *Arid5b^-/-^* cells was decreased relative to the DMSO control cells. However, the OCR of *Arid5b^-/-^* Pre-B cells was still significantly higher compared to Wt cells when treated with BPTES (**Figure 4E**). These results suggest that although glutamine contributes to OxPhos for both Wt and *Arid5b^-/-^* Pre-B cells, it is not responsible for increased OxPhos in *Arid5b^-/-^* Pre-B cells. In the presence of the mitochondrial pyruvate carrier (Mpc1) inhibitor UK5099, which inhibits pyruvate transport into the mitochondria thereby inhibiting its OxPhos, OCR was increased in *Arid5b^-/-^* Pre-B cells indicating that glucose was not responsible for the increased OCR (**Figure 4E**). Additionally, UK5099 treatment did not alter OCR in Wt Pre-B cells suggesting that glucose was not utilized for OxPhos activity (**Figure 4E**). Interestingly, upon treatment with etomoxir, an inhibitor of mitochondrial fatty acid transporter CPT1a, OCR was decreased equally in both Wt and *Arid5b^-/-^* cells (**Figure 4E**). These data indicated that the increased OxPhos in *Arid5b^-/-^* Pre-B cells was fueled primarily by fatty acids, and both Wt and *Arid5b^-/-^* Pre-B cells utilize glutamine and fatty acids but not glucose for OxPhos.

To confirm the increased oxidation of fatty acid in *Arid5b^-/-^* Pre-B cells, we performed palmitate oxidation assays using a Seahorse flux analyzer in Pre-B cells that were cultured in substrate limited media and replenished with palmitate before analysis. As shown in **Figure 4F**, palmitate oxidation was significantly increased in *Arid5b^-/-^* Pre-B cells compared to Wt controls. The elevated fatty acid oxidation suggested increased fatty acid transporters and/or fatty acid uptake into cells. By flow cytometry, we first analyzed CD36 surface expression and uptake of palmitate conjugated with BODIPY in different B lineage cells *ex vivo* in the Wt settings. Both large and small Pre-B cells express the lipid membrane transporter CD36 and consume palmitate; however, in small Pre-B cells both CD36 expression and palmitate uptake decreased significantly compared with large Pre-B cells (**Supplementary Figures 4C, D**). Similarly, in human BM cells, fatty acid uptake peaked at Pre-B I cells (corresponding to large Pre-B in mice) but decreased in Pre-B II (corresponding to small Pre-B in mice) (**Supplementary Figure 4E**). Consistent with increased fatty acid oxidation, palmitate uptake was increased in *Arid5b* ^-/-^ large and small Pre-B cells compared to Wt (**Figures 4G, H**). Furthermore, the expression of fatty acid transporters CD36 was increased in both *Arid5b^-/-^* Pre-B cells compared to the wild type controls (**Figure 4I**).

We recently reported that *Arid5b* deletion in mouse skeletal muscle enhances not only fatty acid metabolism but also glucose uptake which is diverted to the pentose phosphate pathway (PPP) (41). Consistent with these findings, *Arid5b* deletion in Pre-B cells also showed a tendency for increased Glut1 expression and glucose uptake compared to Wt cells (**Supplementary Figures 4F, G**). In addition, the expression of glucose 6 phosphate dehydrogenase (G6pd), the rate limiting enzyme of PPP, was significantly increased in *Arid5b* ^-/-^ Pre-B cells (**Figure 4J**). While *Arid5b* deletion decreases the expression of Mpc1 and Mpc2 in skeletal muscle (41), these proteins were undetectable or very low in Pre-B cells. (**Supplementary Figure 4H**). This indicates an insignificant contribution of pyruvate oxidation in Pre-B cells which is consistent with unaffected OCR values by UK5099 treatment as shown in **Figure 4E** (see also Discussion). These all data indicate that enhanced fatty acid oxidation together with increased PPP is an important prelude for increased cell proliferation of *Arid5b* ^-/-^ Pre-B cells.

### 3.5 Reduced *ARID5B* expression in B cells is associated with leukemic transformation and survival in B-ALL

**Figure 5:**
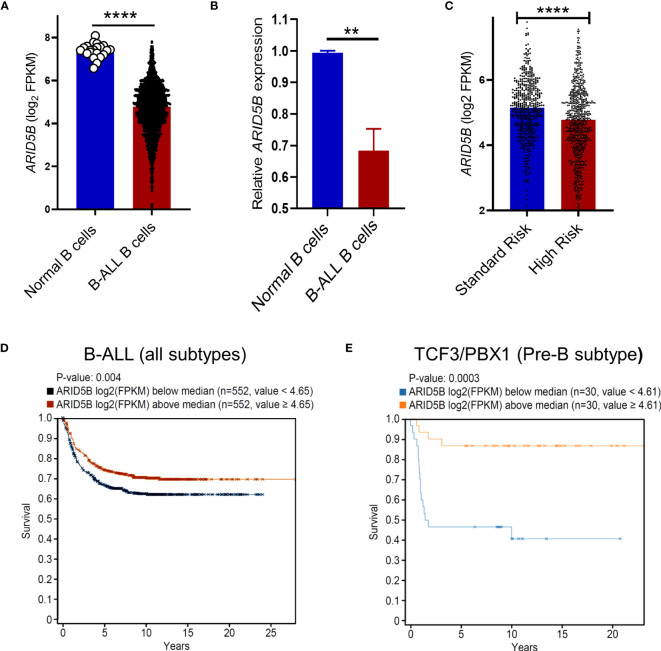
*ARID5B* expression in B cells is associated with leukemic transformation and survival in B-ALL. **(A)** Comparison of *ARID5B* gene expression (FPKM) between human normal CD19^+^B cells (n=21) and leukemic B cells (n=1950). **(B)** *ARID5B* expression (qRTPCR) of CD19^+^ B cells isolated from human apheresis blood (n=3) and CD19^+^ human B-ALL cells Lax7, Lax2, Pdx2. **(C)** *ARID5B* expression in standard vs high risk pediatric B-ALL patients. Clinical data were obtained from Gu et al. (51). **(D)** Kaplan Meier survival curves of B-ALL patients exhibiting *ARID5B* expression above or below the median, n=1104. **(E)** Kaplan Meier survival curve of TCF3/PBX1 B-ALL subtype exhibiting *ARID5B* expression above or below the median n=60. Statistical data **(A-C)** are shown with SEM and were analyzed by Student’s t test. **p < 0.01, ****p < 0.0001.

B cells, particularly at the Pre-B cell stage, are highly vulnerable to leukemic transformation due to rapid proliferation, genetic recombination and elevated metabolism (55). We observed that *Arid5b* deficiency partially blocked B cell differentiation and increased the proliferation of Pre-B cells with enhanced metabolism (**Figures 2** **-** **4**). Additionally, several studies have reported the association of *ARID5B* with B-ALL leukemia risk (31, 32). We propose that reduced *ARID5B* expression in B cells plays a role in Pre-B cell leukemic transformation. To test this, we analyzed publicly available data of 1950 B-ALL patients and 21 human healthy controls from the PeCan database (St Jude). *ARID5B* expression of blasts from B-ALL patients was significantly lower compared to CD19^+^ B cells from healthy controls (**Figure 5A**). By qRT-PCR analysis, we also found decreased expression of *ARID5B* in B-ALL samples compared to normal CD19^+^ B cells isolated from donor apheresis samples (**Figure 5B**). Furthermore, *ARID5B* expression was significantly lower in high-risk pediatric B-ALL patients compared to those of standard risk (**Figure 5C**). In accordance with these data, we also observed a significant increase in mortality of B-ALL patients in which *ARID5B* expression was below versus above the median (**Figure 5D**). We noted that amongst B-ALL patients, the TCF3/PBX1 subtype (Pre-B) (56) demonstrated a much more prominent increase in mortality when *ARID5B* expression was below the median (**Figure 5E**). These data indicate that *ARID5B* expression was not only decreased in leukemic cells but may also influence clinical risk.

In total, these data suggest that low expression of *ARID5B* in B cells is directly associated with leukemogenesis. *ARID5B* is therefore likely to function as a gatekeeper protecting Pre-B cells from leukemic transformation.

## 4 Discussion

Despite an association of *ARID5B* with B-ALL, the fundamental role of *ARID5B* in B cell development has remained largely unexplored. Here, we demonstrated that Arid5b plays an integral role in suppressing proliferation and fatty acid metabolism at the large Pre-B stage and in maintaining quiescence at the small Pre-B stage. Furthermore, we propose reduced *ARID5B* expression plays an important role in B-ALL pathogenesis.

We and others have previously established that *Arid5b* is a developmental regulator in diverse cells types including adipocytes, chondrocytes and muscles (37, 57, 58). Our work, and a very recent study (43), add B cells to the list of cell types whose development is regulated by *Arid5b.* In both mice and humans, *ARID5B* expression was significantly upregulated during B cell development at the Pre-B stage and was maintained onwards indicating a role of *ARID5B* at late stages of B cell development (**Figure 1**). We utilized two approaches to delete *Arid5b*, germline and *ex vivo* Cre-induced deletion, and demonstrated that upregulated *Arid5b* expression plays an essential role in regulating proliferation at Pre-B stages and maintaining quiescence. Since quiescence at the small Pre-B stage is essential for VJ recombination to form a functional BCR, reduced quiescence observed in *Arid5b^-/-^* small Pre-B cells may affect the formation of Ig-kappa light chain which may further impair their differentiation to IgM^+^ immature B cells (8, 20, 21). This may explain why we observed decreased IgM^+^ immature B cells and subsequent B cell populations in lymph nodes and spleen of *Arid5b^-/-^* mice. Our findings of Pre-B cells are in agreement with a recent publication reporting that *Arid5b* overexpression in mouse hematopoietic cells resulted in decreased Pre-B cell populations, whereas *Arid5b* ablation resulted in increased Pre-B cells (43). However, phenotypes in immature and mature B cell populations were found to be different in three types of knockout mice used between the two studies. While we observed decreased immature B cell populations in our 10-15 week old whole-body *Arid5b^-/-^* mice, they observed no differences in 6-8 weeks *Vav1* specific *Arid5b^-/-^* mice but increased immature B cell populations in *Mb1* specific *Arid5b^-/-^* mice. Asides from age differences, these discrepancies in different types of knockout models suggest that *Arid5b* deletion may not only have variable effects during different stages (*Vav1 vs Mb1*) of B cell development but may also be influenced by non-haematopoietic cells in the whole-body knockout model.

Metabolic regulation during B cell development has important consequences on the proliferation of normal and malignant Pre-B cells (4, 5, 59). Although various metabolic pathways have been examined in the context of their contribution to development and leukemogenesis of B cells, there are limited reports about fatty acid metabolism (60). Splenic B cells express fatty acid transporter CD36 (61, 62) and germinal B cells selectively utilize fatty acid oxidation as an energy source while conducting minimal glycolysis (63). However, in bone marrow B cells, no detailed studies have been reported exploring fatty acid metabolism. We uncovered a previously unappreciated phenomenon that fatty acid oxidation contributes a significant component of OxPhos in Pre-B cells in conjunction with glutamine oxidation (**Figure 4E**). In addition, we demonstrated that proliferating B cells in bone marrow (large Pre-B cells in mice and Pre-B I cells in human) express CD36 and consume significant amounts of fatty acid *ex vivo* at a higher level than other stages (**Supplementary Figures 4C-E**). Furthermore, similar to glucose uptake (9–11), our findings imply that fatty acid uptake peaked at large Pre-B stage and then down regulated significantly at quiescent small Pre-B and IgM^+^ immature B cell stages (**Supplementary Figures 4C-E**). Notably, we demonstrated that *Arid5b* regulates this fatty acid uptake and oxidation in Pre-B cells (**Figures 4E-I**). Supporting this phenomenon, our recent study has also showed that *Arid5b* regulates fatty acid oxidation in muscle cells (41).

Glucose is considered one of the major nutrients essential for cellular proliferation and development (59, 64). Through glycolysis and OxPhos, it provides ATP and precursors for nucleic acids and macromolecules. By measuring ECAR and OCR, previous investigations reported that glycolysis and OxPhos increase in the large Pre-B stage and drastically decrease in the small Pre-B cell stage (9, 11). However, those studies did not provide information about the specific substrates fueling the OxPhos. Our data indicated that glucose does not contribute to OxPhos either in Wt or in *Arid5b^-/-^* Pre-B cells, since inhibition of pyruvate transport into mitochondria by the Mpc inhibitor UK5099 did not affect the OCR levels (**Figure 4E**). UK5099 is reported to inhibit the OCR in several types of cells including T cells, plasma B cells and macrophages (65–67). However, in Pre-B cells, we did not observe any effect of this inhibitor on OCR even with increased concentrations (data not shown). This discrepancy raises two possibilities: i) Pre-B cells do not utilize pyruvate for OxPhos, ii) deficit of pyruvate-OxPhos by UK5099 can be compensated by increase in oxidation of fatty acids or glutamine. The first possibility is supported by prior work showing that glucose was not utilized for oxidation in B cells but instead was employed in the biosynthesis of nucleic acids and macromolecules (68). Furthermore, very low or undetectable levels of Mpc1 and Mpc2 expression in Pre-B cells strengthened this likelihood that pyruvate transport to the mitochondria is not activated in Pre-B cells (**Supplementary Figure 4H**). Our study does not deny the contribution of glucose for Pre-B cells proliferation, but rather it suggests that glucose does not go through the pyruvate pathway for OxPhos but possibly shunts to pathways for synthesis of macromolecules and nucleic acids. In agreement with this suggestion, Kojima et al. also showed that glucose deprivation significantly impairs B cells development and is not compensated by pyruvate replenishment (10). These results strongly indicate that glucose is utilized upstream of glycolysis. We analyzed glycolysis in Pre-B cells by measuring ECAR which is predominantly based on the production of lactic acid. Although we did not observe any increase in ECAR and pyruvate mediated OCR, we observed an increase in PPP activity and proliferation in *Arid5b^-/-^* Pre-B cells. Hence, it is likely that the enhanced fatty acid oxidation along with increased expression of PPP observed in *Arid5b^-/-^* Pre-B cells contributes to the anabolic demand for their proliferation. Similar to the observation in Pre-B cells, we found that *Arid5b* deletion in muscles cells enhances PPP (41).

Impaired B cell development driven by *Arid5b* deficiency is likely to impact the leukemic transformation of Pre-B cells. Pre-B cells are susceptible to leukemic transformation because of rapid proliferation, genetic recombination and fluctuating metabolism; therefore, strong gatekeeping mechanisms minimizing the risk of malignant transformation are essential for normal B cell development (26). One major gatekeeping property uniquely possessed by B lineage cells is ‘restrictive/controlled energy metabolism’ which is mediated by transcription factors such as IKZF1, PAX5, EBF1 known to inhibit glucose metabolism (55). We propose that *ARID5B* also functions as a gatekeeper protecting Pre-B cells from malignant transformation based on our findings that i) *Arid5b* deficiency enhances proliferation of Pre-B cells with increased metabolism; ii) *ARID5B* expression is reduced in B-ALL cells compared to normal CD19^+^ B cells; and iii) reduced *ARID5B* expression increases the risk of mortality. The gatekeeping role of *ARID5B* is supported by a bioinformatics study ranking *ARID5B* as one of the top tumor suppressor genes (69). Furthermore, reduced *ARID5B* expression was observed in patients with disease relapse when compared at the time of primary diagnosis of B-ALL (33). Moreover, an *ARID5B* risk allele in B-ALL patients was associated with down regulation of *ARID5B* and mechanistic studies suggest that the risk allele affects the binding of the transcription factors MEF2C and RUNX3 (70, 71). Unlike other genes, *ARID5B* might perform the gatekeeping role by regulating fatty acid metabolism. Indeed, suppression of fatty acid oxidation by the CPT1a inhibitor ST1326 was already shown to reduce the growth of several types of leukemic B cells (72). Taken together, *ARID5B* likely provides protection against the pathogenesis of B-ALL by regulating lipid metabolism.

*Arid5b* is a member of DNA binding protein family which have been described to modulate multiple genes at the transcriptional level (73). We have previously shown that the DNA binding domain of human ARID5B preferentially recognizes an AATA(C/T) core consensus sequence by using electro mobility shift assays (74). This was corroborated in NK cell studies by immunoprecipitation which showed human ARID5B binding to a AATA(C/T) sequence in the promoter region of the ETC complex III component UQCRB thereby impacting oxidative metabolism (39). *Via* a similar mechanism in Pre-B cells, *Arid5b* may regulate the expression of one or many genes involved in multiple signaling and metabolic pathways during the proliferative stage. Our ongoing studies attempt to elucidate the precise molecular mechanisms of how *ARID5B* regulates fatty acid metabolism in Pre-B and leukemic B cells.

We do have some limitations in our study. We were unable to support the increase in *Arid5b* mRNA expression during B cell development with a concomitant increase in protein levels despite several attempts to develop antibody-based detection methods for mouse Arid5b. Nonetheless, our major hypothesis that *Arid5b* plays an important role during B cell development, is still verified from phenotypes observed in *Arid5b* knockout mice. Moreover, although we showed the effect of *Arid5b* deletion on proliferation and metabolism of B cells in BM, additional investigation is warranted to determine if *Arid5b* deletion affects the further B cell maturation in peripheral lymphoid organs as well their immune functions including antibody production and antigen presentation. In addition, we have used BM sorted B220^+^ B cells for *ex vivo* analysis of glycolysis and OxPhos instead of using flow cytometry sorted pure Pre-B cells due to the limited availability of *Arid5b^-/-^* mice and the inherent transient nature of the large and small Pre-B populations (53). Although the majority of cells during the analysis were Pre-B cells (>75%), the observed effect might be contributed not only by the Pre-B population but also by B cells at other stages. Furthermore, due to limited availability of human samples, we could not perform sufficient experiments on human cells to replicate the findings of mice in human cells. Despite these limitations, our current data clearly indicates *Arid5b’s* role in limiting proliferation and fatty acid oxidation during B cell development (**Supplementary Figure 10**). These findings increase our understanding of metabolic regulation during B cell development and shed light on *Arid5b*’s role during B cell development and leukemogenesis.

## Data availability statement

The original contributions presented in the study are included in the article/**Supplementary Material**. Further inquiries can be directed to the corresponding author/s.

## Author contributions

JC designed the study, carried out experiments, analyzed the data, and wrote the manuscript. AE developed the mutant mice, contributed to design experiments and reviewed the manuscript. GL, ML and Y-WH contribute to design experiments, interpret results, critically reviewed manuscript, GZ carried out animal experiments. KI interpreted the results and critically revised the manuscript. All authors contributed to the article and approved the submitted version.

## Supplementary material

Supplementary Figure 1
**(A, B)** *Arid5b* expression in different stages of B cell development in mouse from database GSE100738 **(A)** and Tabula Muris (https://tabula-muris.ds.czbiohub.org/) **(B)**. **(C)** B cell (CD45^+^CD19^+^) population analyzed by flow cytometry at day 12, 18 and 24 during *in vitro* ESC-’B cell differentiation.

Supplementary Figure 2
**(A, B)** Verification of *Arid5b* deletion in BM **(A)** and spleen **(B)** in *Arid5b^-/-^* mice by comparing *Arid5b* mRNA transcript with Wt littermates. *Arid5b* mRNA was analyzed by RTPCR with *beta-Actin* as a normalizer. **(C, D)** T cell population (CD45^+^CD3^+^) and Myeloid cell population (CD45^+^CD11b^+^) in BM and spleen of Wt and *Arid5b^-/-^* mice analysed by flow cytometry. **(E)** Method for *ex vivo Arid5b* deletion from BM B cells from *Arid5b ^fl/fl^* mice by retroviral transduction of Cre. **(F)** Verification flow cytometry plot for transduction efficacy of Cre-ERT2-GFP and Empty-ERT2-GFP plasmids. **(G)** Verification of *ex vivo Arid5b* deletion in BM B cells by qRTPCR. n=3. ****p < 0.0001. Statistical data **(A, B, C, D and G)** are shown with SEM and were analyzed by Student’s t test.

Supplementary Figure 3
**(A, B)** Representative flow cytometry plot of Ki67 analysis for gated large and small Pre-B cells from Wt and *Arid5b^-/-^* mice. **(C, D)** Cell cycle analysis by flow cytometry which were calculated based on DAPI staining of Cre-induced *Arid5b* deleted cells *ex vivo*. The experiment was repeated three times with similar results. **(E-K)** mRNA expression of *Myc, Myb, Foxo1, Blnk, Ikzf1, Foxo3a and Fnip1* in sorted large Pre-B and small Pre-B cells from Wt and *Arid5b^-/-^* mice. n=3. Statistical data are shown with SEM and were analyzed by Student’s t test *p < 0.05, ** p < 0.01

Supplementary Figure 4
**(A)** Representative flow cytometry analysis of Pre-B cell populations after 72 hrs culture of B220^+^ B cells in presence of IL-7. This analysis was performed immediately prior to Seahorse metabolic assays and repeated several times with similar results. **(B)** OCR by Seahorse analysis in *ex vivo* Cre-induced *Arid5b* deleted B cells cultured in presence of IL-7 for 72 hr. Representative seahorse trace of OCR analysis. n=4 in each group. Experiment was repeated three times with similar results. **(C)** Histogram of CD36 MFI expression analysed by flow cytometry in Pro-B cells (CD19^+^cKIT^+^BP1^-^IgM^-^), large Pre-B cells (CD19^+^BP1^+^cKIT^-^IgM^-^FSC^high^) and small Pre-B cells (CD19^+^BP1^+^cKIT^-^IgM^-^FCS^low^) and Immature B cells (CD19^+^IgM^+^BP1^-^cKIT^-^) from Wt mice. **(D)** Palmitate uptake in different B cell subsets of Wt mice. BM B cells from mice were cultured for 72 hr in presence of IL-7 and further incubated with 1 µM green fluorescent palmitate-BODIPY (FL C16) for 3 hr followed by flow cytometry analysis. Shown are the bar graph (left) for average (MFI) of palmitate BODIPY uptake and representative histograms (right) in different subsets of B cells. n=4. **(E)** Bar graph (left) for average palmitate-BODIPY uptake in different subsets of human cells: Pro-B cells (CD10^+^CD19^-^CD34^+^), Pre-B I cells (CD10^+^CD19^+^CD34^+^), Pre-B II cells (CD10^+^CD19^+^CD20^+^CD34^-^IgM^-^) and Immature B cells (CD10^+^CD19^+^CD20^+^IgM^+^CD34^-^). Cells were analyzed as described in **(C)**. Histogram (right side) are representative flow cytometry MFI data. n=3. **(F)** Glut1 expression (MFI) calculated from flow cytometry in large and small Pre-B cells from the mouse BM cells which were cultured for 72 hr in presence of IL-7, n=4. **(G)** Glucose uptake in BM cells which were isolated from Wt and *Arid5b^-/-^* mice and cultured for 72 hr in presence of IL-7, n=6. **(H)** Mpc1 and Mpc2 protein expression by western blot in Wt and *Arid5b^-/-^* Pre-B cells. Statistical data are shown with SEM and were analyzed by Student’s t test *p < 0.05, ** p < 0.01, *** p < 0.001, ****p < 0.0001.

Supplementary Figure 5 Flow cytometry gating strategy for Pro-B cells (B220^+^CD19^+^cKIT^high^IgM^-^), total Pre-B cells (B220^+^CD19^+^BP^+^IgM^-^), large Pre-B cells (B220^+^CD19^+^BP^+^IgM^-^FSC^high^), small Pre-B cells (B220^+^CD19^+^BP^+^IgM^-^FSC^low^), Immature B cells (B220^+^CD19^+^IgM^-^IgD^-^) and recirculating B cells (B220^+^CD19^+^IgM^-^IgD^+^) from mouse BM.

Supplementary Figure 6 Flow cytometry gating strategy for HSC (Lin^-^Sca^+^CKIT^+^CD34^+^CD150^+^) and CLP (Lin^-^Sca^+^cKIT^+^CD34^+^IL-7RA^+^) in mouse BM.

Supplementary Figure 7 Flow cytometry gating strategy for B lineage cells analysis for different B cells subsets of human BM. Pro-B cells (CD10^+^CD19^-^CD34^+^), Pre-B I cells (CD10^+^CD19^+^CD34^+^), Pre-B II cells (CD10^+^CD19^+^CD34^-^CD20^+^IgM^-^) and Immature B cells (CD10^+^CD19^+^CD20^+^IgM^+^CD34^-^).

Supplementary Figure 8 Flow cytometry gating strategy for B lineage cells analysis in *ex vivo Arid5b* deleted B cells by retroviral transduction of Cre.

Supplementary Figure 9 Flow cytometry gating strategy for sorting of Pro-B cells (CD10^+^CD19^-^CD34^+^) and total Pre-B cells (CD10^+^CD19^+^IgM^-^) from human BM.

Supplementary Figure 10 Schematic diagram for role of *Arid5b* during B cell development: *Arid5b* expression is upregulated at the Pre-B cell stages and is subsequently maintained, and plays a role in limiting proliferation and lipid metabolism.
