# Supplementary figures and images for: ARID5B regulates fatty acid metabolism and proliferation at the Pre-B cell stage during B cell development
Source: Front Immunol. 2023 Jul 7;14:1170475. doi: 10.3389/fimmu.2023.1170475 (PMC10360657; doi:10.3389/fimmu.2023.1170475)

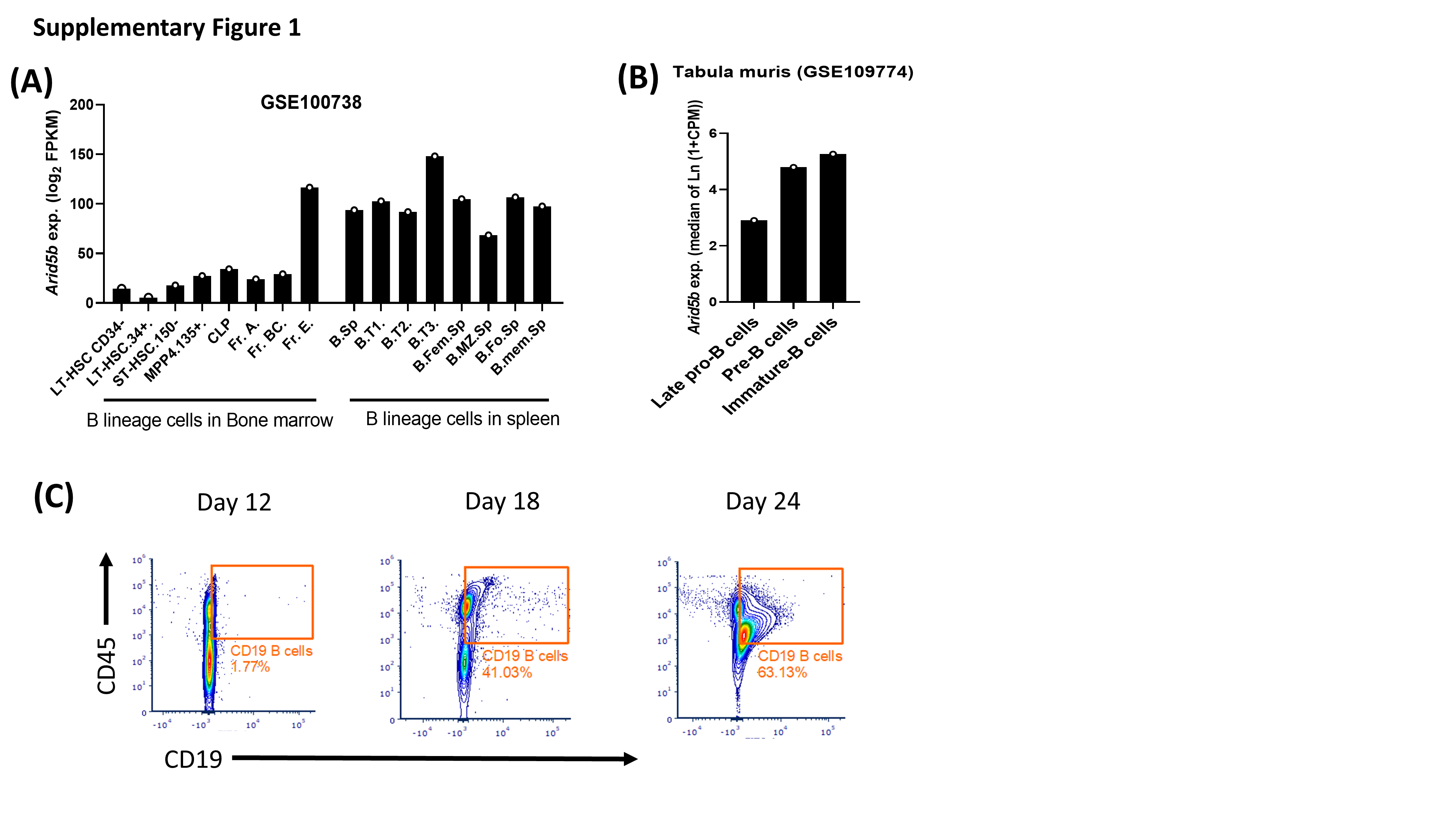

Supplement: Supplementary Figure 1 — (A, B) Arid5b expression in different stages of B cell development in mouse from database GSE100738 (A) and Tabula Muris (https://tabula-muris.ds.czbiohub.org/) (B). (C) B cell (CD45+CD19+) population analyzed by flow cytometry at day 12, 18 and 24 during in vitro ESC-’B cell differentiation. [file Image_1.tif]

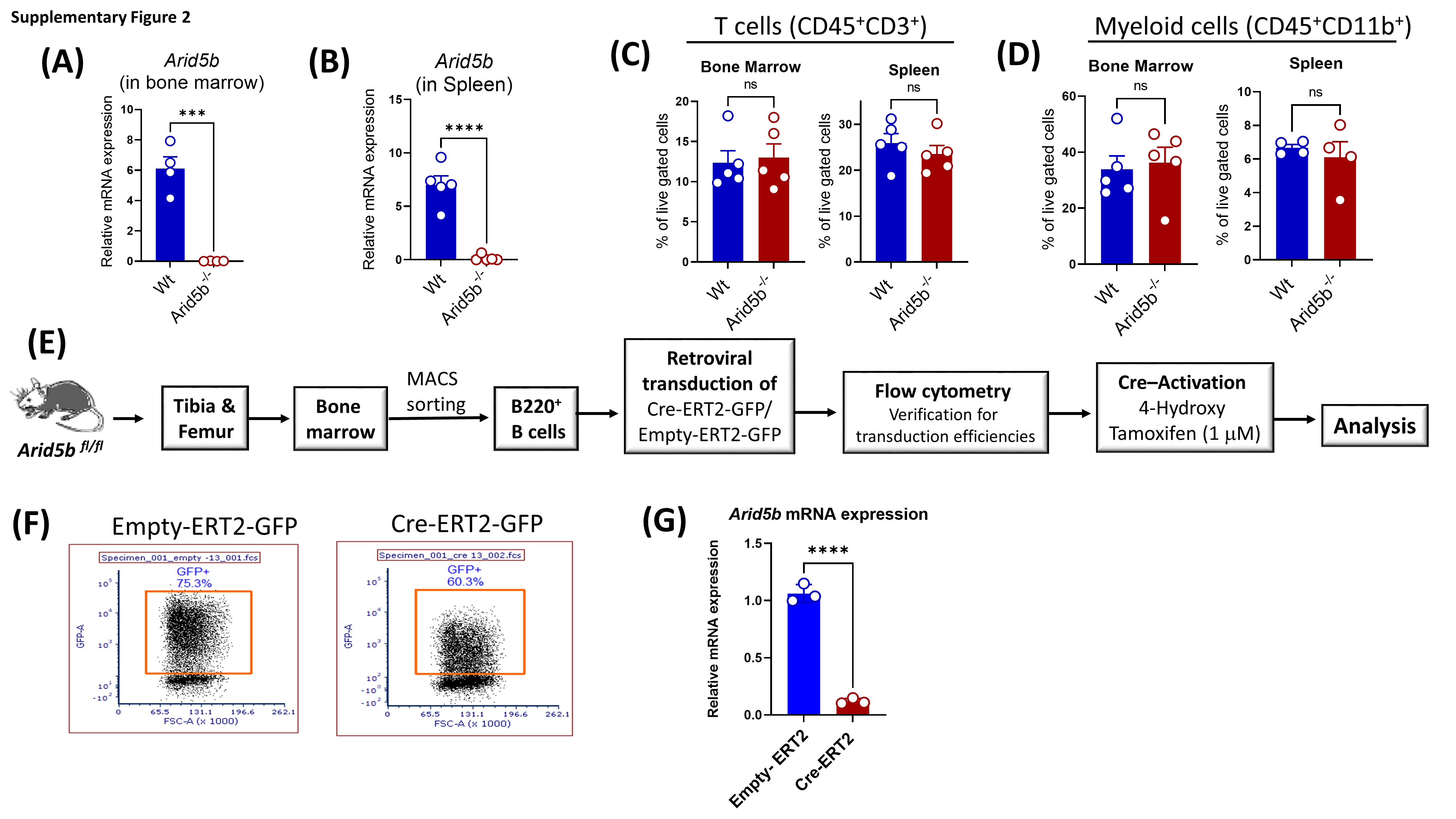

Supplement: Supplementary Figure 2 — (A, B) Verification of Arid5b deletion in BM (A) and spleen (B) in Arid5b-/- mice by comparing Arid5b mRNA transcript with Wt littermates. Arid5b mRNA was analyzed by RTPCR with beta-Actin as a normalizer. (C, D) T cell population (CD45+CD3+) and Myeloid cell population (CD45+CD11b+) in BM and spleen of Wt and Arid5b-/- mice analysed by flow cytometry. (E) Method for ex vivo Arid5b deletion from BM B cells from Arid5b fl/fl mice by retroviral transduction of Cre. (F) Verification flow cytometry plot for transduction efficacy of Cre-ERT2-GFP and Empty-ERT2-GFP plasmids. (G) Verification of ex vivo Arid5b deletion in BM B cells by qRTPCR. n=3. ****p < 0.0001. Statistical data (A, B, C, D and G) are shown with SEM and were analyzed by Student’s t test. [file Image_2.tif]

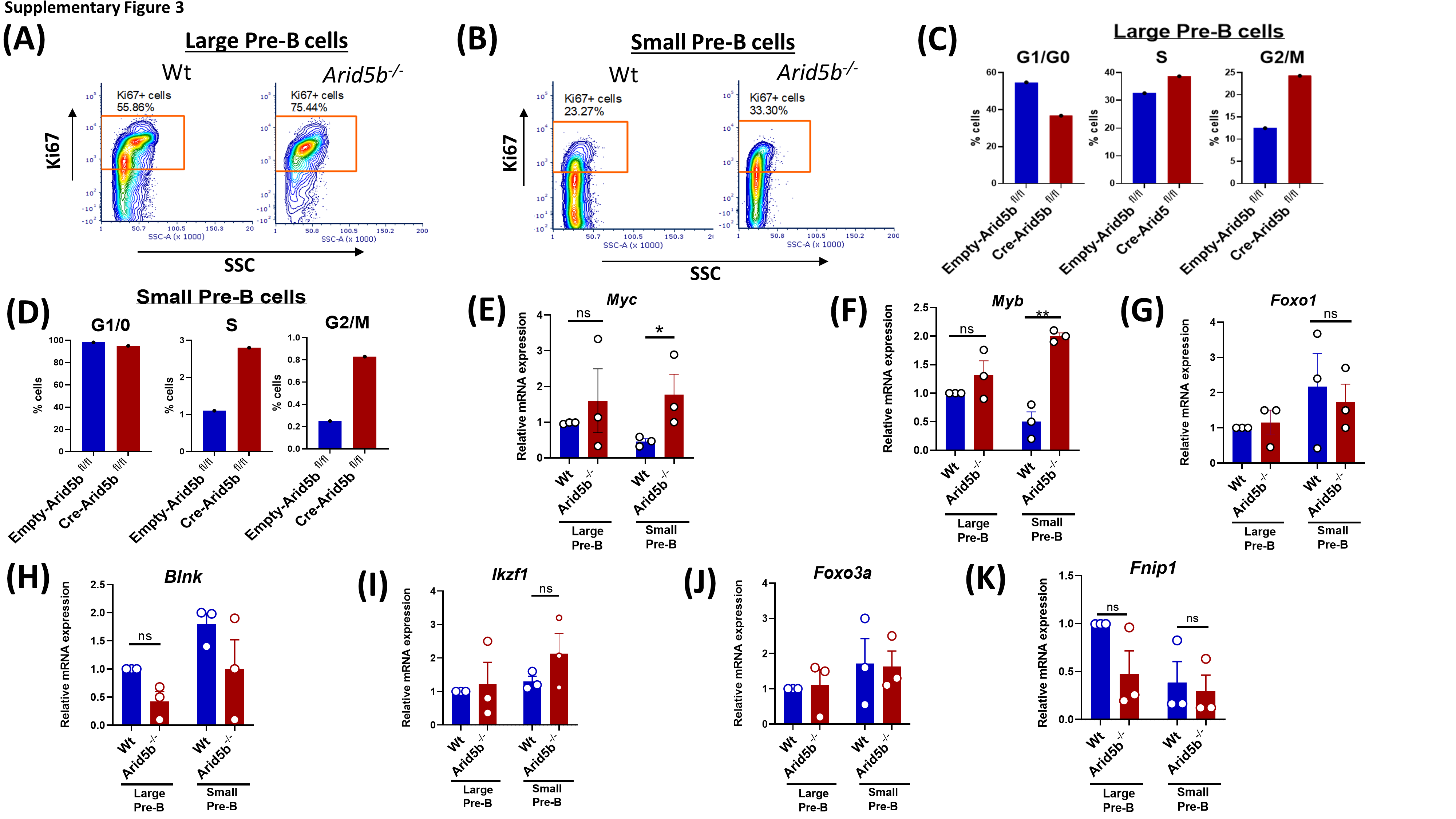

Supplement: Supplementary Figure 3 — (A, B) Representative flow cytometry plot of Ki67 analysis for gated large and small Pre-B cells from Wt and Arid5b-/- mice. (C, D) Cell cycle analysis by flow cytometry which were calculated based on DAPI staining of Cre-induced Arid5b deleted cells ex vivo. The experiment was repeated three times with similar results. (E-K) mRNA expression of Myc, Myb, Foxo1, Blnk, Ikzf1, Foxo3a and Fnip1 in sorted large Pre-B and small Pre-B cells from Wt and Arid5b-/- mice. n=3. Statistical data are shown with SEM and were analyzed by Student’s t test *p < 0.05, ** p < 0.01 [file Image_3.tif]

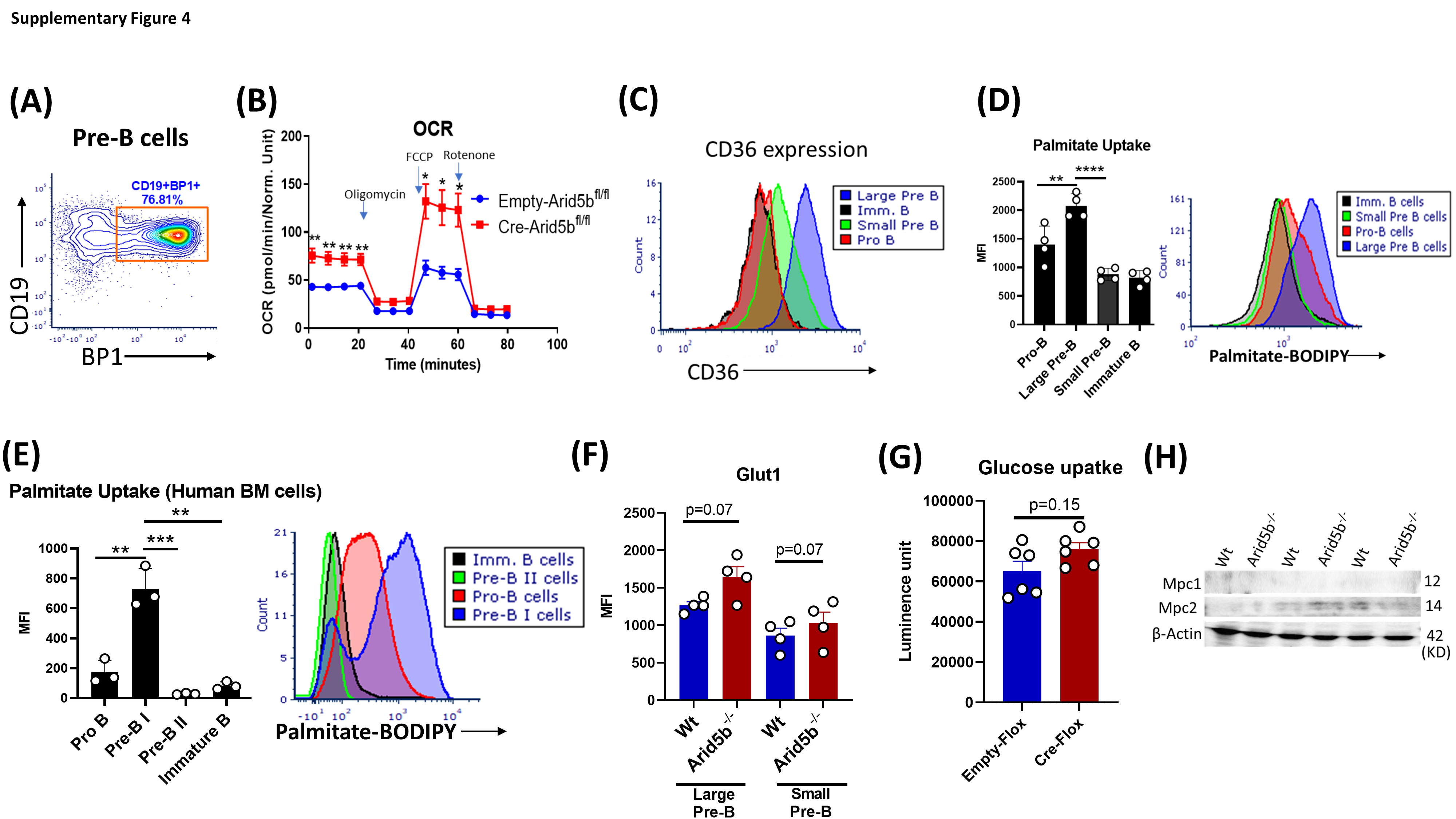

Supplement: Supplementary Figure 4 — (A) Representative flow cytometry analysis of Pre-B cell populations after 72 hrs culture of B220+ B cells in presence of IL-7. This analysis was performed immediately prior to Seahorse metabolic assays and repeated several times with similar results. (B) OCR by Seahorse analysis in ex vivo Cre-induced Arid5b deleted B cells cultured in presence of IL-7 for 72 hr. Representative seahorse trace of OCR analysis. n=4 in each group. Experiment was repeated three times with similar results. (C) Histogram of CD36 MFI expression analysed by flow cytometry in Pro-B cells (CD19+cKIT+BP1-IgM-), large Pre-B cells (CD19+BP1+cKIT-IgM-FSChigh) and small Pre-B cells (CD19+BP1+cKIT-IgM-FCSlow) and Immature B cells (CD19+IgM+BP1-cKIT-) from Wt mice. (D) Palmitate uptake in different B cell subsets of Wt mice. BM B cells from mice were cultured for 72 hr in presence of IL-7 and further incubated with 1 µM green fluorescent palmitate-BODIPY (FL C16) for 3 hr followed by flow cytometry analysis. Shown are the bar graph (left) for average (MFI) of palmitate BODIPY uptake and representative histograms (right) in different subsets of B cells. n=4. (E) Bar graph (left) for average palmitate-BODIPY uptake in different subsets of human cells: Pro-B cells (CD10+CD19-CD34+), Pre-B I cells (CD10+CD19+CD34+), Pre-B II cells (CD10+CD19+CD20+CD34-IgM-) and Immature B cells (CD10+CD19+CD20+IgM+CD34-). Cells were analyzed as described in (C). Histogram (right side) are representative flow cytometry MFI data. n=3. (F) Glut1 expression (MFI) calculated from flow cytometry in large and small Pre-B cells from the mouse BM cells which were cultured for 72 hr in presence of IL-7, n=4. (G) Glucose uptake in BM cells which were isolated from Wt and Arid5b-/- mice and cultured for 72 hr in presence of IL-7, n=6. (H) Mpc1 and Mpc2 protein expression by western blot in Wt and Arid5b-/- Pre-B cells. Statistical data are shown with SEM and were analyzed by Student’s t test *p < 0.05, ** p < 0.01, *** p < 0.001, [file Image_4.tif]

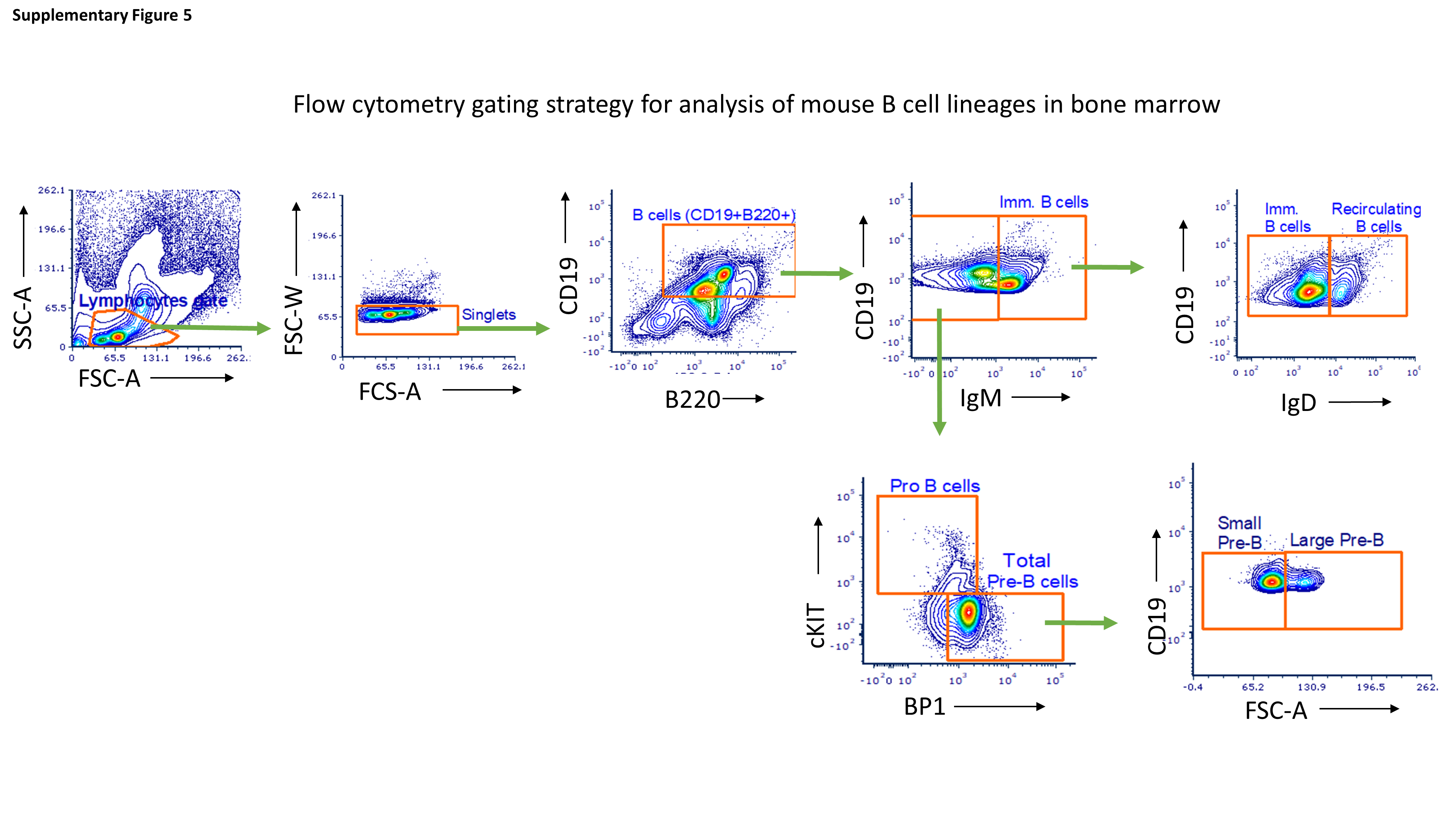

Supplement: Supplementary Figure 5 — Flow cytometry gating strategy for Pro-B cells (B220+CD19+cKIThighIgM-), total Pre-B cells (B220+CD19+BP+IgM-), large Pre-B cells (B220+CD19+BP+IgM-FSChigh), small Pre-B cells (B220+CD19+BP+IgM-FSClow), Immature B cells (B220+CD19+IgM-IgD-) and recirculating B cells (B220+CD19+IgM-IgD+) from mouse BM. [file Image_5.tif]

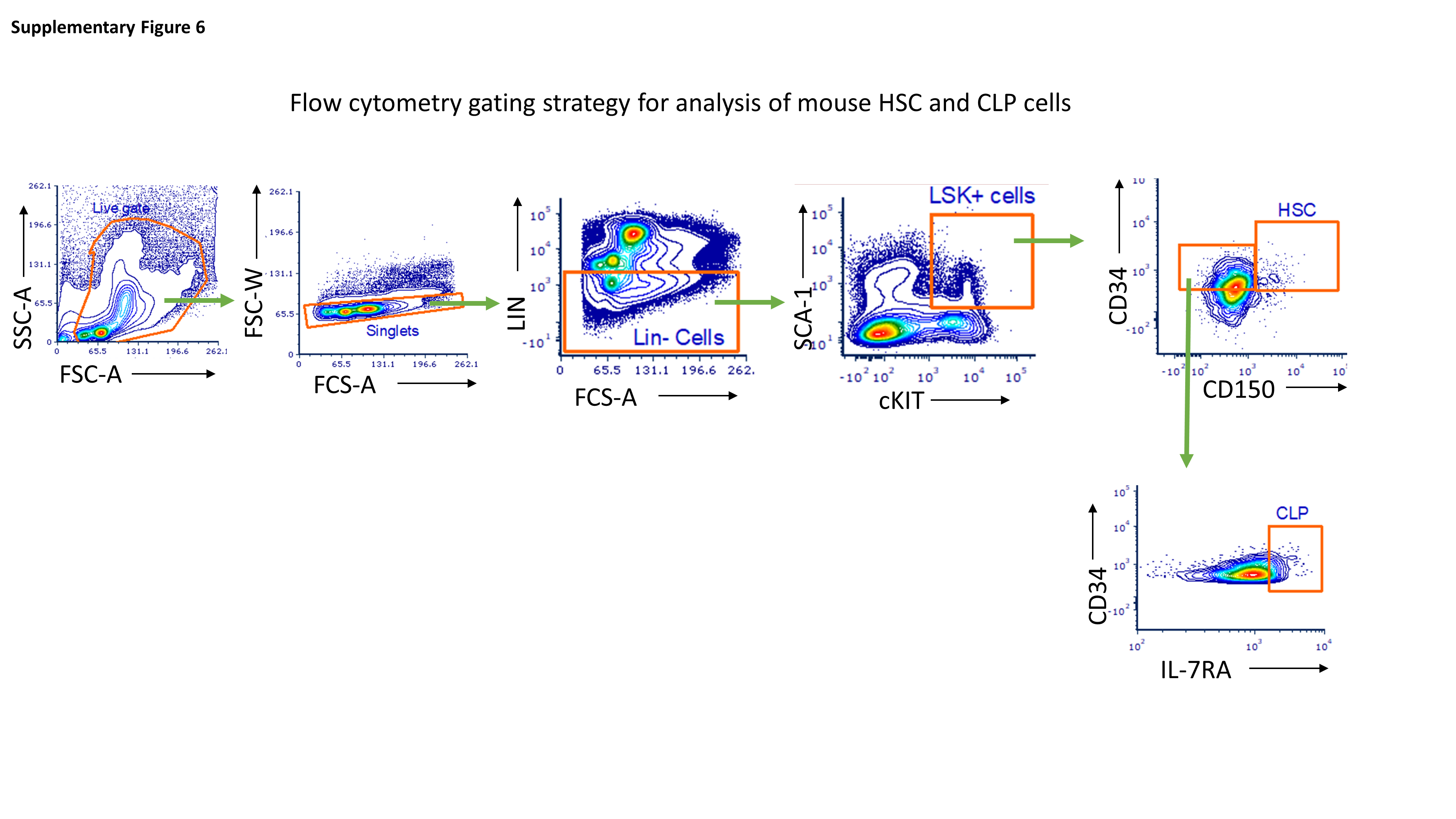

Supplement: Supplementary Figure 6 — Flow cytometry gating strategy for HSC (Lin-Sca+CKIT+CD34+CD150+) and CLP (Lin-Sca+cKIT+CD34+IL-7RA+) in mouse BM. [file Image_6.tif]

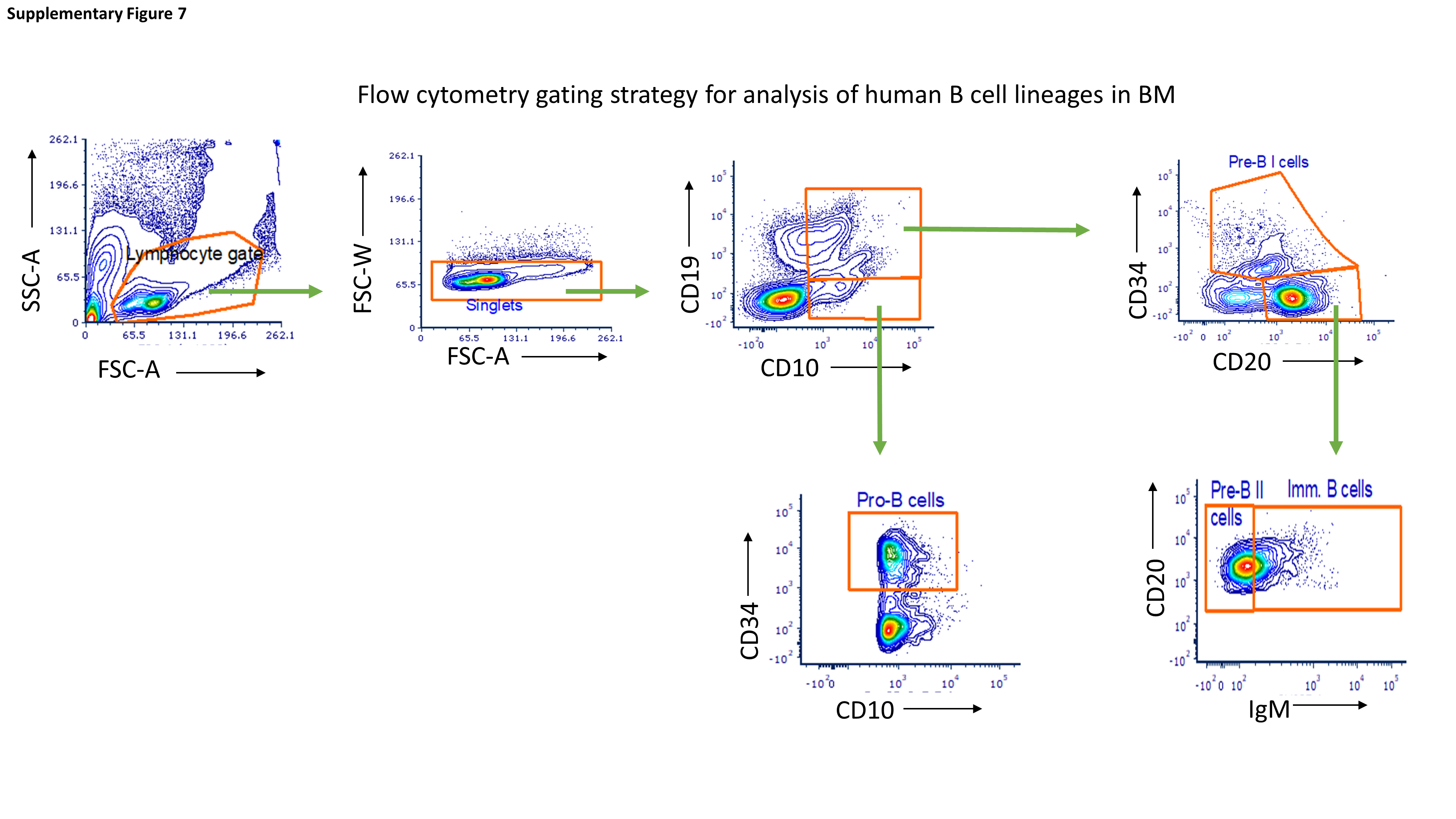

Supplement: Supplementary Figure 7 — Flow cytometry gating strategy for B lineage cells analysis for different B cells subsets of human BM. Pro-B cells (CD10+CD19-CD34+), Pre-B I cells (CD10+CD19+CD34+), Pre-B II cells (CD10+CD19+CD34-CD20+IgM-) and Immature B cells (CD10+CD19+CD20+IgM+CD34-). [file Image_7.tif]

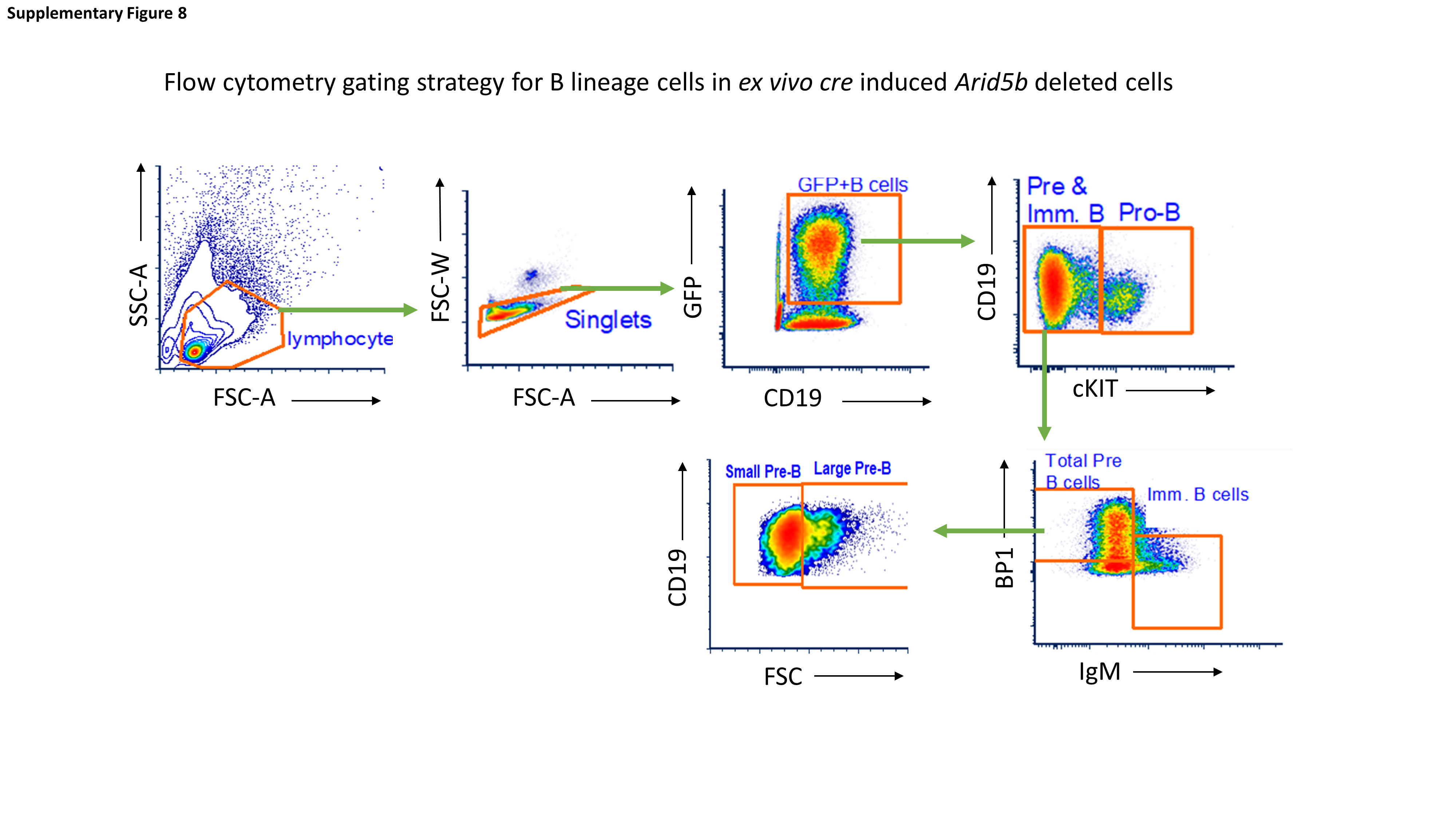

Supplement: Supplementary Figure 8 — Flow cytometry gating strategy for B lineage cells analysis in ex vivo Arid5b deleted B cells by retroviral transduction of Cre. [file Image_8.tif]

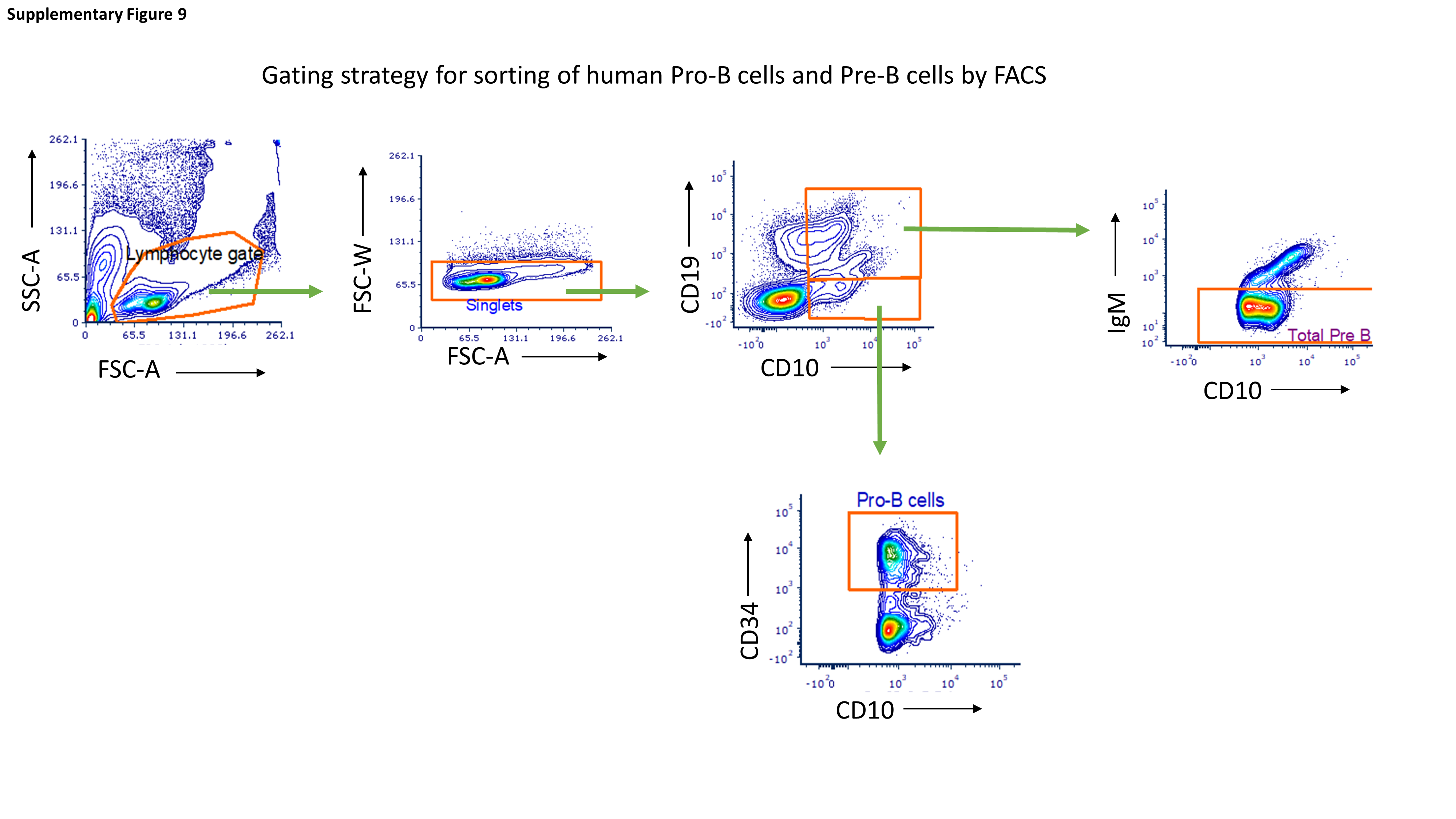

Supplement: Supplementary Figure 9 — Flow cytometry gating strategy for sorting of Pro-B cells (CD10+CD19-CD34+) and total Pre-B cells (CD10+CD19+IgM-) from human BM. [file Image_9.tif]

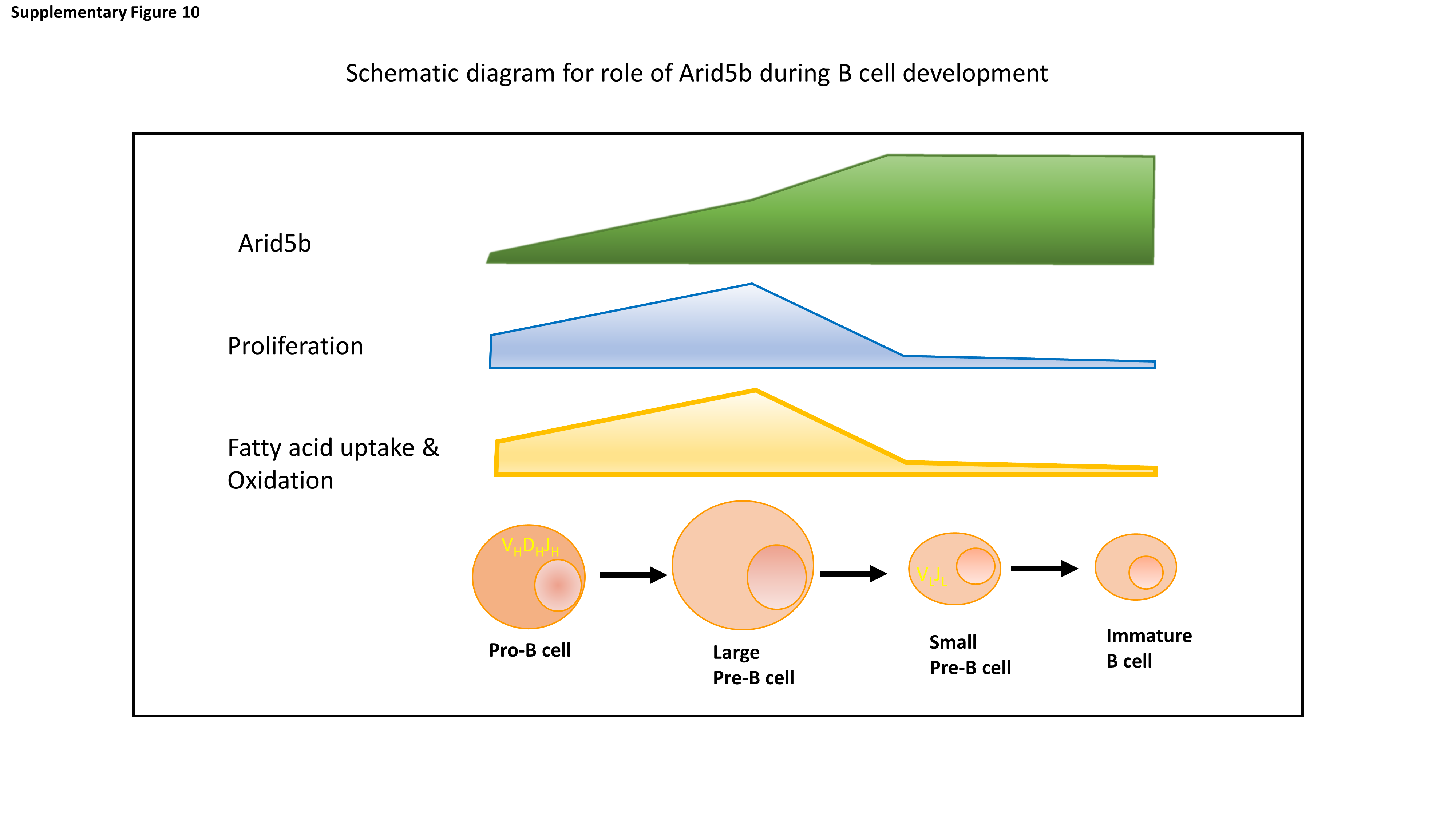

Supplement: Supplementary Figure 10 — Schematic diagram for role of Arid5b during B cell development: Arid5b expression is upregulated at the Pre-B cell stages and is subsequently maintained, and plays a role in limiting proliferation and lipid metabolism. [file Image_10.tif]
