# Supplementary material for: ARID5B regulates fatty acid metabolism and proliferation at the Pre-B cell stage during B cell development
Source: Front Immunol. 2023 Jul 7;14:1170475. doi: 10.3389/fimmu.2023.1170475 (PMC10360657; doi:10.3389/fimmu.2023.1170475)
Supplement: Supplementary file 11 [file Table_1.pdf]

frontiers

Supplementary Table 1: Antibodies used for flow cytometry

**Antibody name**	**Clone**	**Host species**	**Manufacturer**
Anti-mouse CD19	6D5	Rat	Biolegend
Ani-mouse B220	RA3-6B2	Rat	Biolegend
Anti-mouse IgM	RMM-1	Rat	Biolegend
Anti-mouse BP1	6C3	Rat	Biolegend
Anti-mouse cKIT	2B8	Rat	Biolegend
Anti-mouse CD150	TC15-12F12.2	Rat	Biolegend
Anti-mouse Sca-1	D7	Rat	Biolegend
Anti-mouse CD34	HM34	Armenian Hamster	Biolegend
Anti-mouse CD36	HM36	Armenian Hamster	Biolegend
Anti-mouse Glut1	--	Rabbit	Novus Biologicals
Anti-mouse Ki67	16A8	Rat	Biolegend
Anti-mouse IgLC-kappa	RMK-45	Rat	Biolegend
Anti-mouse CD43	S11	Rat	Biolegend
Anti-mouse IL-7RA	A7R34	Rat	Biolegend
Anti-human CD10	HI10a	mouse	Biolegend
Anti-human CD19	4G7	mouse	Biolegend
Anti-human CD20	2H7	mouse	Biolegend
Anti-human CD34	581	mouse	Biolegend
Anti-human IgM	MHM-88	mouse	Biolegend
