# Supplementary material for: ARID5B regulates fatty acid metabolism and proliferation at the Pre-B cell stage during B cell development
Source: Front Immunol. 2023 Jul 7;14:1170475. doi: 10.3389/fimmu.2023.1170475 (PMC10360657; doi:10.3389/fimmu.2023.1170475)
Supplement: Supplementary file 12 [file Table_2.pdf]

Supplementary Table 2: Primers for qRTPCR

**Target**	**Manufacturer's ID**
Mouse *Arid5b*	Mm01148813_m1
Mouse *Myc*	Mm00487804_m1
Mouse *Myb*	Mm00501741_m1
Mouse *Fnip1*	Mm00620486_m1
Mouse *Ikzf1*	Mm01187878_m1
Mouse *Foxo1*	Mm00490671_m1
Mouse *Foxo3a*	Mm01185722_m1
Mouse *Beta Actin*	Mm04394036_g1
Mouse *Glut1*	Mm00449511_m1
Mouse *Blnk*	Mm01197846_m1
Human *ARID5B*	Hs01382781_m1
Human *BETA-ACTIN*	Hs01060665_g1
